# Pip shape echoes grapevine domestication history

**DOI:** 10.1038/s41598-021-00877-4

**Published:** 2021-11-01

**Authors:** Vincent Bonhomme, Sarah Ivorra, Thierry Lacombe, Allowen Evin, Isabel Figueiral, David Maghradze, Cécile Marchal, Clémence Pagnoux, Thierry Pastor, Hervé Pomarèdes, Roberto Bacilieri, Jean-Frédéric Terral, Laurent Bouby

**Affiliations:** 1 ISEM, University of Montpellier, CNRS, EPHE, IRD, Montpellier, France; 2 UMR AGAP Institut, Univ Montpellier, CIRAD, INRAE, Institut Agro, 34398 Montpellier, France; 3 Grapevine Biological Resources Center, INRAE, Unité Expérimentale Domaine de Vassal, University of Montpellier, Marseillan, France; 4 INRAP Méditerranée, Center of Villeneuve-les-Béziers, Villeneuve-les-Béziers, France; 5 National Wine Agency of Georgia, Tbilisi, Georgia; 6 École Française d’Athènes, Athens, Greece

**Keywords:** Plant domestication, Plant evolution

## Abstract

The pip, as the most common grapevine archaeological remain, is extensively used to document past viticulture dynamics. This paper uses state of the art morphological analyses to analyse the largest reference collection of modern pips to date, representative of the present-day diversity of the domesticated grapevine from Western Eurasia. We tested for a costructure between the form of the modern pips and the: destination use (table/wine), geographical origins, and populational labels obtained through two molecular approaches. Significant structuring is demonstrated for each of these cofactors and for the first time it is possible to infer properties of varieties without going through the parallel with modern varieties. These results provide a unique tool that can be applied to archaeological pips in order to reconstruct the spatio-temporal dynamics of grape diversity on a large scale and to better understand viticulture history. The models obtained were then used to infer the affiliations with archaeobotanical remains recovered in Mas de Vignoles XIV (Nîmes, France). The results show a twofold shift between the Late Iron Age and the Middle Ages, from table to wine grape varieties and from eastern to western origins which correlates with previous palaeogenomic results.

## Introduction

Today, grapevine (*Vitis vinifera* L. subsp. *vinifera*) is economically one of the most important cultivated fruit species in the world^1^. Its central economic and cultural role in the Mediterranean Basin goes back beyond the Greco-Roman era^2^. Modern genetics and archaeobotany concur in locating the origin of domesticated grapevine in the Near East, south of the Caucasus^3,4^. Its initial domestication is thought to have occurred during the Neolithic (between 6000 and 3000 BC) but the date is still debated. Chemical analyses of pottery vessels suggest that wine was already produced in the Caucasus area 8000–6000 years ago^2,5^. From its Near-Eastern cradle, viticulture spread to most of the Mediterranean and eventually the rest of modern-day Europe, between 3000 BC and 500 CE^2^. Viticulture could have started in Sardinia and Southern Italy as early as the late 2nd millennium BC^6^ and in Southern Spain by the beginning of the 1st millennium BC, in connection with the Phoenician influence^7^.

Grapevine has been dramatically modified and diversified since its early domestication. The most notable changes concern: the shift from dioecy in wild grapevines (*Vitis vinifera* subsp. *sylvestris*) to a hermaphroditic reproductive system for most of the varieties, the increase in berry and bunch sizes, the increase in sugar and acid content, and the variation in berry colour and shape^8,9^. These changes are so significant that the phenotypic diversity of the domestic grapevine, including its morphological component, is much greater than that of its wild counterpart^8^. Several thousand varieties can be distinguished^10^, and are generally classified in two main groups: table (fruits consumed fresh or dried) and wine grapes.

Cultivated grapevine diversity is patrimonial and a direct product of its intertwined history with human societies. Because of this, cultivar diversity can help understand this shared history through the use of genetic or morphological markers.

This paper explores the global grapevine diversity through the analysis of seed morphology. It is known that seeds from wild and domesticated grapes differ in their form (i.e. size and shape); wild grapes produce roundish pips with short stalks and cultivated varieties produce more elongated pips with longer stalks^8^. Grape pips have long been a focal point in archaeobotanical studies, because of these well-known differences and because they often are the only remains that are preserved in archaeological contexts. Morphometrics, or the statistical description of shape, has a prominent place in the quantitative analysis of pips. Morphological characterization works on a highly integrated and well preserved datum, the pip shape, and its capacity to signal phenotypic resemblances, give major insights into domestication studies using modern and ancient material^11–14^.

Various pip measurements have been used to distinguish wild and domesticated *V. vinifera* subspecies in the archaeological record^15,16^. Recent quantifications of pip shape that use outline analyses have helped to improve the discrimination towards the identification of individual varieties^17–22^.

The study of the whole phenotypic diversity in cultivated varieties reveals that two clear types can be discerned: table and wine varieties^23^. Table grapes tend to have large berries, sometimes seedless, and have relatively thin skin while wine grapes are smaller, have higher concentrations of sugar, are generally seeded, and have relatively thick skins^24^.

Negrul^25–27^ proposed a comprehensive classification of all the known varieties into three major groups or *proles*. *Proles orientalis* is composed of table grape varieties, with big berries, typical of the Near and Middle East and regions of the Mediterranean basin. *Proles occidentalis* gathers wine grape varieties from Central and Western Europe. They are typically more resistant to low temperatures and have smaller, more acidic berries with lower sugar content. The varieties of *Proles pontica* (Balkans, Black Sea and Caucasus) have intermediate characteristics between *orientalis* and *occidentalis*. They are mostly used for winemaking or for both table/wine purposes.

In recent years, the global diversity of cultivated grapevines has mostly been studied with the use of nuclear microsatellite markers^28–30^, thousands of SNP markers^3,30,31^, or both^32^. These studies tend to identify a global structure confirming the three major groups described by Negrul^25^, as well as an additional group of Iberian varieties, and a number of varieties with admixed/intermediate assignment.

Using 20 microsatellite markers (SSR), Bacilieri et al.^30^ were able to identify Negrul’s three major groups, and a second level with two additional groups: “Iberian Peninsula and Maghreb” and “Table grapes from Italy and central Europe”. In Lacombe’s study^29^, four distinct groups could be recognized using the same markers on a reduced set of varieties, in which closely related genotypes were excluded. In the fourth group, the Iberian cultivars were found to be associated to wine and table varieties from Asia Minor and the Caucasus. Laucou et al.’s findings^31^, which were based on an array of 18k single nucleotide polymorphisms (SNP), present a similar organization into four main groups, with the Iberian and Negrul’s groups, however, the majority of varieties were admixed.

This global structuring of *V. vinifera*, determined by its predominant use by humans and geographic origin, is a result of the long history of grape domestication and of the spread of viticulture. Negrul’s hypothesis proposes that the main groups of cultivars were domesticated from different populations of wild grapevines^25^. The existence of secondary domestication events with local wild populations as opposed to mere introgression processes in other areas of the Mediterranean is still discussed, and may have helped shaped regional diversity^3,33,34^. The modern diversity of cultivated grapes stems from thousands of years of selection and diffusion through cuttings and seeds combining spontaneous hybridization and somatic variation^31^.

Following morphometric analyses of grape leaves which demonstrate a weak correlation between leaf morphology and the East/West origin of grape varieties^35^, we decided to use seed outline analysis to explore the structure of the diversity of cultivated varieties across the entire Eurasian and Mediterranean area. Our research aims to establish solid foundations on modern material, to further fuel archaeobotanical studies that can provide insights into past grapevine diversity and viticulture history. We used a representative collection of modern grapevine diversity in the form of a photographic pip shape collection, and we tested the reliability of discriminant models based on shape to infer: (i) destination use, (ii) geographical origins, (iii) conformity in the genetic structure found among varieties. Finally, we applied these models on archaeological remains as a first step into drawing finer-grained, morphological-based inferences about viticulture in the past.

## Materials and methods

### Reference collection of modern pips

This study includes 434 grapevine modern cultivars (Table A ESM). Their origins cover the entirety of Euro-Mediterranean diversity. Most of the cultivars were selected and sampled from the INRAE Grape Germplasm Repository (Marseillan-Plage, France). Additionally, autochthonous cultivars from the Caucasus area were sampled from the Saguramo Grape Repository (Jighaura, Georgia).

For each cultivar, 30 normally developed berries were randomly collected from a single, fully-ripe bunch. The final dataset comprised 12,346 pips.

Cofactors further used are presented in the Table A (ESM) and summarised in the Table B (ESM). They comprised: berry size, geographic origins, and destination use assessed from general bibliography^10,36^. We also included genetic assignation^30,31^. These two studies and the present one largely used the same set of varieties. However, because these sets were compiled at different time and with different aims, information may be missing, debated or unknown, for any given variety (Table A). For our analyses, only cultivars with well-defined information were used (Tables A, B). Berry size was observed and recorded over several years in the grapevine repositories and coded according to the International Organization of Vine and Wine descriptors^37^.

### Archaeological material

As a case study, we selected the site of Mas de Vignoles XIV (Nîmes, France) where large quantities of well-preserved, waterlogged, grape pips dating back to two time-periods (Late Iron Age/Early Roman and Medieval times) were found in association with other plant remains.

The site is located in the alluvial plain of the river Vistre and was excavated by INRAP^38^. The site was occupied at different periods between the Neolithic and the early Middle Ages. The first traces of occupation are very sporadic, but from the end of the Bronze Age onwards, the area appears to undergo continuous changes in land use, mainly oriented towards agricultural and craft activities and including few traces of human habitation. During the second century BCE (late Iron Age or Republican period) a large farmhouse was identified nearby; the remains of its northern boundary were uncovered at Mas de Vignoles XIV. This farmhouse was later replaced by two small farms surrounded by areas devoted to agriculture and animal husbandry. The pips from this period were recovered from a well (PT14203, SU14258) and a ditch (FO14194, SU14152) that was part of a network of ditches delimiting the farming areas.

Archaeological remains from the Middle Ages are not abundant, nor well preserved and include very few traces of human habitation; this suggests that people lived further away, probably due to the unfavourable topography and edaphic conditions (depression area with high water table). The archaeological structures found include several ditches, wells, storage pits, animal enclosures and wooden constructions dedicated to farming activities ; the importance of animal husbandry is suggested by the abundance of cattle remains, which is unusual in a region mainly dominated by sheep/goat, but in agreement with local conditions^39^. The presence of beetles associated with stable areas further reinforce the evidence of animal husbandry. Flax and hemp figure among the potentially cultivated plants, other than grapevine. The *Vitis* pips investigated come from a well (PT12024, SU12109 and 12111) radiocarbon-dated to the Early Middle Ages: Poz-48697: 1200 ± 30 BP (706–945 cal AD^40^).

Nine pips from Mas de Vignoles XIV previously delivered aDNA results showing that varieties of different origins may have been cultivated during the Late Iron Age and the Middle Ages^41^.

### Pip morphometric description

Each pip was photographed according to two orthogonal views (dorsal and lateral) by the same operator (TP). Outline coordinates (x; y) were extracted from these images and two markers (one at each tip of the pips) were used to normalize the position, size, rotation and first point of the outlines by registering them on “Bookstein coordinates”, that are (x = − 0.5; y = 0) and (x = 0.5; y = 0) coordinate points. For each view, elliptical Fourier transforms were used to convert the contour geometry into “Fourier coefficients”. Elliptical Fourier transforms are detailed elsewhere^42,43^. The number of harmonics was chosen to gather 95% of the total harmonic power^43^, which corresponds to five for both views. In terms of operator error (e.g. while positioning the pip), this is less harmonics, and thus a conservative choice, compared to previous recommendations of six harmonics for both views^22^. With four coefficients per harmonic, 40 coefficients were obtained and further used as quantitative variables describing the shape. Pip length was derived from outline coordinates. Pip length was shown to be the best predictor of all other lengths measured on pips^20^ and here helped to analyse form, that is the shape plus size. When compared to manual measurements obtained in a subset of another study^20^, error was centred and was, on average, below 1% (~ 1/20 mm). The correlation between the berry and the pip size previously shown^20^ was here tested on a larger dataset using one-tail Wilcoxon rank tests (medium vs. small, large vs. small; Fig. A ESM). The final matrix analysed and used in models was thus [12346 × 41].

### Statistical environment

Analyses were performed in R 4.0.2^44^, with the packages Momocs 1.3.2^43^ for everything morphometrics, MASS 7.3-51.6^45^ for linear discriminant analyses, tidyverse 1.2.1^46^ for general data manipulation and visualization, pvclust 2.2-0^47^ for assessing uncertainties in hierarchical clustering and ape 5.0^48^ for unrooted tree representation.

### Visualizing and testing for use, geographical and genetic signals

First, a principal component analysis was calculated on the full matrix of coefficients to visualize how each level of each cofactor of interest were located in this synthetic morphological space (Fig. B ESM).

To test for a costructure between pip form and cofactors, two approaches were used: hierarchical clustering with robustness assessment, and cross-validation using permutational and balanced linear discriminant analyses. To assess geographical structuration the (putative) countries of origin of cultivars were organized in geographical groups (Table A ESM, Table B ESM).

**Figure 1:**
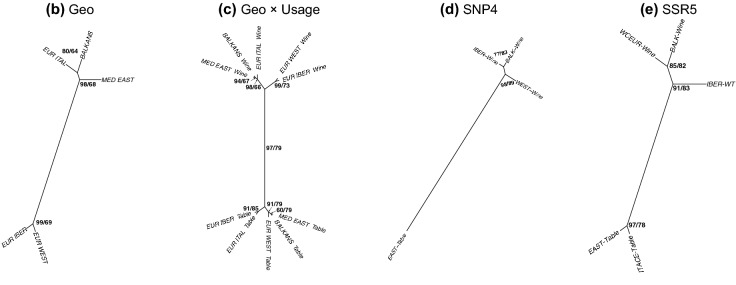
Unrooted trees obtained with hierarchical clustering of the form of pips (length + shape) grouped according to covariates of interest. For each node the numbers correspond to pvclust/cross-validation values. Tree (**a**) corresponding to use, as a two-class case has no topology and is not presented.

For hierarchical clustering, the averaged coefficients for each level were used to calculate a distance matrix using correlation as the distance method, on which a hierarchical clustering using average (i.e. UPGMA) was calculated. Topologies obtained were presented as unrooted trees (Figs. 1, C ESM). The robustness of nodes was estimated through multiscale resampling^47^ with proportions ranging from r = 0.5 to r = 1.4 with a 0.1 increment and 10^2^ resampling with replacement. The “approximately unbiased p-values” were retained^49^ and presented in Figs. 1 and C (ESM).

**Figure 2:**
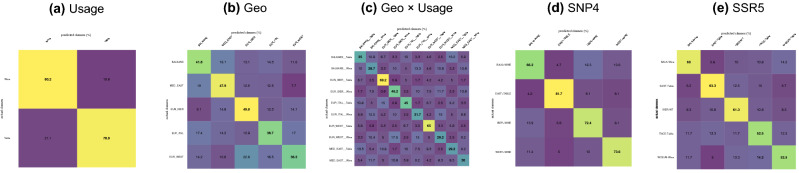
Confusion matrices for discriminant analyses. Cells present median percentages obtained over 100 permutations of balanced datasets. Along the diagonal, values in bold indicate significant values (i.e. above the maximal value obtained by chance alone among 100 permutations).

This method had the merit of simplicity but did not accounted for: (a) variability within groups, (b) overlapping between groups, and (c) unbalanced groups sizes since coefficients were averaged. Moreover, for archaeobotanical inference, we need predictive models along with their performance assessment. We thus combined this approach with linear discriminant analyses (LDA). Class accuracies (i.e. tree leaves) were presented as confusion matrices, and class clustering (i.e. tree nodes) were estimated using classes belonging to a node against all others. The index used is accuracy (the proportion of correctly classified pips), using leave-one-out cross-validation. To cope with unbalanced sample sizes, 10^2^ permutations were used^50^, and each sampled the minimal group size among all groups. The median value was reported on each node in Fig. 1. For each model, we also presented the confusion matrices obtained for each group against others (Fig. 2), as well as the distribution of accuracies obtained under this null model (here obtained through simulations but expected to follow a multinomial distribution; Fig. D ESM).

### Assessing filtering predictions

In a “predictive” LDA, new statistical individuals are always assigned to the class with the highest posterior probability. In filtering out predictions based on posterior probabilities sample size is exchanged for identification confidence. The posterior probability cut-off value is often based on rule of thumb by picking an arbitrary threshold. Here we use a twofold approach, combining LDA, resampling and filtering in the spirit of^50^. Across the 10^2^ permutations, we calculated the class accuracies and the proportion of the original group sample size retained, as functions of cut-off value for posterior probability (Fig. E ESM and Table C ESM). We also explored the same relationship using the proportion of cases among permutations where each pip was attributed to a given class as a cut-off value (Fig. E ESM and Table D).

### Inferences on archaeological material

**Figure 3:**
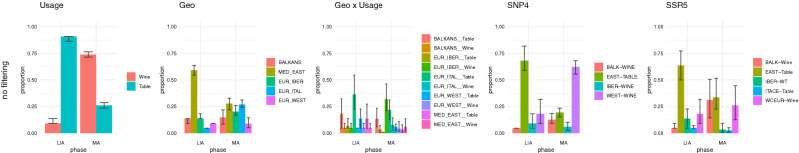
Inferences for archaeological pips of the domesticated type from the Mas de Vignoles XIV. The columns correspond to the different models presented after an inference on the wild/domesticated. The proportion are presented without filtering (see also Fig. F ESM).

Former studies have shown that archaeobotanical assemblages generally include an important proportion of wild type grape pips^16,22^. For this reason, we used a first LDA_status_ to identify domesticated and wild type pips with the dataset published^17,51^. Here, we used the same approach with 10^2^ balanced permutations. For each pip, the majority rule applied. In the cited studies, these LDA achieved 95% accuracy (without filtering) in distinguishing between pips from wild grapevine individuals and those from domesticated varieties. Further inferences about archaeological pips identified as the domesticated type were then obtained using the 10^2^ balanced models previously presented (Figs. 3 and F ESM): we inferred destination use (LDA_Use_), geographical origins (LDA_Geo_), the destination and geographical origins jointly (LDA_Geo×Use_) and genetical grouping (LDA_SNP4_, LDA_SSR5_). Inferred proportions of each class are presented with three approaches: no filtering (Fig. 3), filtering out pips with a median posterior probability observed among permutations < 0.8 (Fig. F ESM); filtering out pips that were attributed to the same class less than 50% of the time (Fig. F ESM).

## Results

### Berry size

Our results show that berry size in modern grapes positively correlates with pip size (Fig. A ESM): varieties with medium-sized berries had longer pips than those with small-sized berries (Wilcoxon rank tests: W = 6,581,329, P < 10^–16^). Similarly, large-sized berries had longer pips than those with medium-sized berries (W = 11,851,551, P < 10^–16^).

### Principal component analysis

The first two components of the PCA obtained from the full matrix of coefficients captured 73.2% of the total variance in form. For the sake of clarity, for each variety only the PC1-PC2 centroid was displayed (Fig. B ESM). With the exception of “Use”, which shows a clear positional difference between table and wine varieties, other cofactors of interests showed more subtle contrasts.

### Form and destination use

After discarding mixed destination varieties, the remaining set included 3,106 pips (Table B ESM). The discriminant model achieved a good discrimination rate (wine = 80.2%; table = 78.9%—Fig. 2), far better than the results expected of chance alone (expected = 50%; max. observed for 100 permutations = 53%—Figs. 2 and D ESM).

### Form and geographical origin

The first geographical model, based on the putative origin of cultivars according to our bibliography, included all regions except NEW WORLD. For each group, 480 pips were included. The resulting tree (Fig. C ESM) showed a clear geographical structuring with two separate clusters (EUR_IBER + EUR_WEST; EUR_ITAL + BALKANS + MED_SOUTH + MED_EAST) with EUR_EAST in between. Varieties gathered in the ASIA_CENT group were clearly set apart. For the other nodes, pvclust values were all > 94 and cross-validation > 64%. Class accuracies ranged between 23% (MED_EAST) and 63% (ASIA_CENT); aside from MED_EAST, all were better than chance alone (expected = 12.5%; max_100_ = 25%).

A second geographical model was restricted to “core” historical regions (Fig. 1). In practical terms, the varietal sampling within these groups was more exhaustive and allowed to include 1,439 pips in permutations (Table B ESM). The resulting tree (Fig. 1) clearly distinguished between EUR_IBER + EUR_WEST and the other groups. All nodes presented pvclust values > 80 and cv values > 64%. Class accuracies ranged between 36% (EUR_WEST) and 50% (EUR_IBER), all better than chance alone (expected 20%; max_100_ = 29%—Figs. 2 and D ESM).

With fewer groups, one usually expects better accuracies; since this was not the case, we suspected a latent effect. We thus built a third model including the same core geographical regions, and combined them with their destination use (Fig. 1). Due to the lower number of table varieties, only 120 pips were included in each permutation (Table B ESM). Despite this, a clear structure with two neat clades corresponding to destination use were observed. Apart from the clade representing BALKANS_Table and MED_EAST_Table, all nodes had pvclust values > 91 and cv values > 66% (Fig. 2). For the wine varieties, BALKANS and MED_EAST were clustered together (pvclust = 94; cv = 67%), then with ITAL (pvclust = 98; cv = 66%). Among wine varieties, another clade grouped EUR_WEST and EUR_IBER (pvclust = 99; cv = 73%). Class accuracies ranged between 29% (BALKANS_Wine, EUR_WEST_Wine, MED_EAST_Table) and 69% (EUR_IBER_Table), overall, far better than chance alone (expected = 10%; max_100_ = 22%—Figs. 2 and D ESM).

### Form and genetic structure

We then explored whether the genetic structure found using SSR^30^ and SNP^31^ data was also echoed in pip form. The first model used SNP4 (Fig. 1) and included 360 pips in each permutation. The EAST_TABLE group was distinguishable from the three other groups (pvclust = 98; cv = 89%), and in the latter BALK_Wine and IBER_Wine clustered together (pvclust = 77; cv = 83%). Cross-validation values for each node were all > 83%. Class accuracies for each group ranged from 66% (BALK_Wine) to 82% (EAST_Table), much better than chance alone (expected = 25%; max_100_ = 38%—Figs. 2 and D ESM).

The second model used microsatellite data (SSR5) and led to similar results. This model used only 120 pips for each permutation and distinguished two groups EAST_Table and ITACE_Table, and WCEUR_Wine + BALK_Wine + IBER_WT. All nodes presented pvclust values > 85 and cv values > 78% (Fig. 1). Class accuracies ranged from 53% (ITACE-Table and WCEUR-Wine) to 63% (EAST-Table), again, better than chance alone (expected = 20%; max_100_ = 35%—Figs. 2 and D ESM). We built a final model excluding ITACE_Table (not shown), which allowed us to increase the number of pips to 420. The same topology was obtained among remaining groups, all pvclust values were > 77 and cv values > 70%, and nodes and class accuracies for groups ranged from 67 to 71%, much better than chance alone (expected = 25%; max_100_ = 36%).

### Sample size and filtering out based on posterior probabilities

As expected, filtering results based on posterior probabilities improves class accuracies at the cost of reduced sample sizes (Fig. E ESM, Tables C ESM and D ESM). The models with the lowest class accuracies before any filtering were the ones with the steepest slopes for the proportion of filtered out curves. Such simulations are useful since they show that with low class accuracies and without filtering on posterior probabilities, the benefit in the accuracy gain is quite low compared to the price to pay in terms of sample size reduction. For instance, when filtering at a posterior probability of 0.5, the absolute gain is only 11% (33% of relative gain) but 64% of the original sample size is filtered out. Also, the models with the smallest number of pips in each permutation showed the highest uncertainties for class accuracy estimates. Due to the resampling nature of these simulations, the higher the number of pips, the lower the variation expected for the estimates.

### Application to the archaeological material

LDA_status_ trained on the reference material led to 95.2% accuracy for both wild and domesticated types (100 permutation using 2005 pips). When applied to archaeological material, LDA_status_ classified 81% pips (102/128) and 39% (74/204) pips as the wild morphological type for the Late Iron age (LIA) and the Middle Age (MA) phases. These pips were discarded from further analyses and the remaining sample sizes were thus 26 and 130 for LIA and MA phases. Overall, the different filtering approaches led to congruent results (Figs. 3, F ESM). We tried a high pass of 0.8 for posterior probabilities whose results on archaeological material (not shown) confirmed those obtained on modern material: sample sizes were dramatically reduced and results were much more dependent on the training set. The overall tendencies observed for the Mas de Vignoles XIV and based on pip shape were a shift from table to wine type, and from Southwest Asia to Western Europe, between the LIA and MA. The cofactors including use in their definitions (Use, Geo × Use, SNP4 and SSR5) all corroborated the predominance of the table type during the LIA and of the wine type during the MA. Similarly, for the geographical origins, all models provide evidence of the Eastern origins for the LIA and of Western European origins for the MA (Figs. 3, F ESM). The length of the pips classified in the domesticated-type of the LIA assemblage is greater than that of the MA. Consequently, the berry size that can be inferred is higher for the LIA, close to medium-size to large modern berries, while the size inferred for MA berries is very small (Fig. A ESM).

## Discussion

The destination use (table/wine) and geographical origins of *V. vinifera* are echoed in the shape of modern grapevine pips and corroborate the structure found using genetic markers. The results here obtained from this modern material dataset pave the way for a more comprehensive archaeobotanical analysis of the grapevine historical agrobiodiversity and biogeography.

Analyses of genomic sequences brought direct insights into *V. vinifera* genealogies, kinships and, more generally, into the intraspecific structuring of the domesticated grapevine^3,4,30,31,52,53^. Genetic markers are direct, sensitive and accurate proxies, but do not provide clear-cut groups within agrobiodiversity since variety amelioration is the product of a continuous and intertwined history and are rarely performed on the large spatio-temporal scale offered by morphometric studies. The two studies where genetic assignation were used demonstrate a high proportion of admixed varieties^30,31^.

Pip shape, on the other hand, integrates genotypic, developmental and environmental factors. As a phenotypical trait, it is known to be a much more indirect proxy for measuring and uncovering agrobiodiversity structuring. Because morphology is prone to homoplasy, two identical shapes may not be directly genetically related and may instead reflect a potentially mixed signal of ancestry, similar environmental adaptations, as well as non-adaptive natural processes (i.e. drift). Our results are validated by molecular approaches rather than *confirming* them. Under certain conditions, for example where a strong population structure and divergent selection are present, phenotypical approaches may be superior to molecular ones for measuring agrobiodiversity^54^.

Our results show that grapevine pip diversity is significantly structured by use and, to a lesser extent, by the geographical origins of varieties. Use had the best class accuracies; the pips of wine and table grapevine varieties have different form. This was shown in a previous study, which used a smaller set of varieties^20^. Several other studies based on phenotypic^23,25^ and genetic markers^3,30,31^, have already concluded that grape varieties were structured, above all, according to their use as table or wine.

More importantly, our results also established a significant geographical correlation in the shape of pips. Between the two genetic models, the classifications from SNP4 gave better class accuracies for pips than those of SSR5. The fact that SNP4 was calculated using 10,000 SNP markers scattered along each chromosome, bolsters the findings of our study since the SSR5, only used 20 microsatellite markers. Despite having one class less, the SNP4 dataset may be more representative of pip variability since it was trained using more varieties, and thus more pips in permutations.

The large-scale structure in pip shape reflects the same blurred boundaries as those reported by genomic analyses. In genetic analyses, unassigned varieties are mostly attributed to human-assisted movement of cultivars across regions and inter-group breeding. In morphologic analysis, however, additional factors may be involved. i.e. environment and development constraints as well as homoplasy. Nevertheless, the morphology-based geographical tree indicates the clustering of eastern groups and western groups, and Italian varieties clustering with eastern ones. The same pattern is found for the tree combining geography and use, where despite a predominant Use structure, the Italian wine varieties are clustered with eastern ones.

Interestingly, incorrect assignations may also reveal meaningful information. Misclassified seeds fall primarily into groups that are closely related in terms of use and geographical origin. For instance, in the Geographical confusion matrix, the eastern Mediterranean group is most frequently misclassified with pips of the “Balkans” groups, and vice-versa. On the same confusion matrix, the “EUR-ITAL” group reflects its intermediate nature between western and eastern varieties. For these reasons, retrieving congruent results using shape alone was far from a foregone conclusion.

Finally, it is worth noting that the *proles* classification proposed by Negrul^25^ was based on morphological criteria and was later confirmed by genetical studies^30,31^. These different approaches are largely congruent and provide evidence that grapevine diversity is not only structured, but that its structuring is related to, and likely a product of, the history and the geography of viticulture.

Dedicated genotype-to-phenotype association studies could help decipher the mechanisms behind such correlation between the shape of pips and the cofactors of destination use and geographical origins. The destination use is directly related to the phenotypic traits and chiefly those of the berry (e.g. size, flavors, aromas, etc.). Berry trait loci have been reported in other studies^32,55^, as have the covariation between the berry and the pip size and shape^20^. With regard to geographical origins, we cannot exclude an indirect link with climatic conditions through crossing of varieties from different origins but we see no reason why a particular geographical origin may directly select a particular pip shape. Berry size likely has a correlation with geographical origin because of the relationship between use and geography. Negrul^25^ already highlighted the ubiquity of large-berry table varieties in the Near-Middle East, that of small-berry wine varieties in Central-Western Europe, and intermediate varieties in his *proles pontica* (Balkans, Caucasus, Black Sea). Overall, it is likely that pip shape was not directly implicated in selection and that the subtle changes in its shape are probably neutral. One can thus reasonably hypothesize that any shape change is therefore caused by genetic drift and/or genetic linkage.

While indirect, both in origin and signature, pip shape is informative about variety origins and use. Shape is also often the only exploitable datum on archaeological remains, and these results are therefore of prime interest to help us better understand grapevine agrobiodiversity through both time and space^16,17,21^.

Archaeobotanical inference is mostly actualistic: insights obtained from modern material generate inferences for archaeological remains. So far, the shape of pips has been used to distinguish between wild and domesticated types^15,16,22^ and, more recently, to identify domesticated morphotypes that correlate with modern varieties^17,21^. The identification of similar modern varieties amongst a diverse subset can be used to make infraspecific conclusions if their properties are compared to those of the already identified varieties^21,51^.

In our study, we used a more extensive and more representative collection of modern seeds, the largest worldwide, to the best of our knowledge. The pioneer collection used in Terral et al.^22^ increased since then^17,51,56^. Here, we moreover directly infer cofactors of interest without the intermediate identification of the most-resembling modern variety. This is an important result because it means that properties of varieties cultivated in the distant past can be inferred directly from the archaeological remains without having to compare them with modern varieties that may not be appropriate counterparts.

To increase the robustness of inferences, predictions are sometimes filtered out based on posterior probabilities^12,51^. When cut-off thresholds are selected by the “rule of thumb” method, the trade-off between accuracy and sample size is often forgotten. Our results indicated that this approach should be used sparingly and only when classification accuracies were already proven to be better than classification by chance alone. One should also keep in mind that poorly classified pips may actually be “true” intermediate forms, and if filtered out, may accentuate the contrast between or within assemblages. Finally, when using linear discriminant analyses, particularly when groups have significantly unbalanced sample sizes, the use of permutations is preferable for obtaining better estimates of their accuracies^50^.

We applied the models trained on modern material to the Mas de Vignoles XIV assemblages whose palaeogenomic information identifying the relationships between ancient grapes and modern diversity had already been obtained^41^. It is an ideal opportunity to combine aDNA approaches with and morphometrics strike force, although not on the same pips.

Our morphometric analyses of the Mas de Vignoles XIV assemblage demonstrate notable changes in viticulture between the Late Iron Age and Middle Age. The first one was the decreased prevalence of the wild type, from a predominant to a minority proportion. This decrease was previously observed in the study of several other archaeological sites in southern France^16^. It can be assumed that this wild type, which is phenotypically different from modern varieties, corresponds to a part of the diversity amongst historically cultivated varieties rather than being gathered wild berries^16^.

Changes were also observed in domesticated pips. The different models agree on a twofold trend “between” the LIA and the MA: a shift from table to wine varieties, and from eastern to western varieties. These changes are probably two facets of the same shift. The pip lengths are also much smaller for the Middle Age assemblages. This direct measure is congruent with the LDA results as well as those of a previous study, which showed that wine varieties have shorter pips than table ones^20^. The SSR4 model, that should be considered more robust than the SSR5 as discussed above, similarly exhibits a contrast between these two historical phases: the predominance of Eastern table varieties in the LIA and of Western wine varieties in the MA. According to aDNA results, the three seeds studied from the LIA sample resemble table grapes as well as Eastern and Iberian wine varieties whereas medieval seeds are more similar to Western European wine varieties^41^. Although the seeds used were not the same as those of our study, they nevertheless corroborate our observations of a geographical shift towards Western wine varieties.

It is not possible to prove with certainty that the grape seeds found at Mas de Vignoles XIV came from locally grown grapevines, but its rural location may suggest this. While grapes were traded and could be transported over long distances in Roman times^57^, archaeological findings have revealed traces of grapevine cultivation on the outskirts of the city of Nîmes, in the close vicinity of Mas de Vignoles XIV, from the second century BC onwards^58^. Until recently, it was believed that by the late Iron Age the vines cultivated in the South of France were only intended for wine production^59^. However, while vineyards appeared to be fairly extensive around the city of Nîmes during the early Roman period, wine cellars and wine production equipment were not very widespread in the excavated settlements^58^. This observation could be consistent with the hypothesis that parts of vineyards were dedicated to the cultivation of table grapes. These new results allow us to imagine the possibility of a viticulture partly destined for table purposes using eastern varieties rather than native or locally domesticated varieties.

## Supplementary Information

Supplementary Information 1.

Supplementary Information 2.

Supplementary Information 3.

Supplementary Information 4.

Supplementary Information 5.

Supplementary Information 6.

Supplementary Information 7.

Supplementary Information 8.

## Data Availability

Full datasets will be released upon acceptance. They are (privately) available there: https://figshare.com/s/7578a0740dfbac9e3742.
