# Supplementary material for: Pip shape echoes grapevine domestication history
Source: Sci Rep. 2021 Nov 1;11:21381. doi: 10.1038/s41598-021-00877-4 (PMC8560759; doi:10.1038/s41598-021-00877-4)
Supplement: Supplementary file 1 — Supplementary Information 1. [file 41598_2021_877_MOESM1_ESM.docx]

**ELECTRONIC SUPPLEMENTARY MATERIAL FOR**

## Pip shape echoes grapevine domestication history

Vincent Bonhomme^1,*^, Sarah Ivorra^1^, Thierry Lacombe^2, 3,^, Allowen Evin^1^, Isabel Figueiral^1, 4^, David Maghradze^5^, Cécile Marchal^3^, Clémence Pagnoux^1, 6^, Thierry Pastor^1^, Hervé Pomarèdes^4^, Roberto Bacilieri^2^, Jean-Frédéric Terral^1^ and Laurent Bouby^1,*^

^1^ ISEM, University of Montpellier, CNRS, EPHE, IRD, Montpellier, France

^2^ AGAP, University of Montpellier, CIRAD, INRAE, Institut Agro, Montpellier, France

^3^ Grapevine Biological Resources Center, INRAE, Unité Expérimentale Domaine de Vassal, University of Montpellier, Marseillan, France

^4^ INRAP Méditerranée, Center of Villeneuve-les-Béziers, France.

^5^ National Wine Agency of Georgia, Tbilisi, Georgia

^6^ École française d’Athènes, Athens, Greece

*corresponding authors

**Email**

bonhomme.vincent@gmail.com, laurent.bouby@umontpellier.fr

**(provided as .pdf)**

**Table A ESM**: List of varieties included in this study. Mean pip length is presented with ± 1SD. For geographical origins, NA denotes debated or unknown putative origins; for SNP4 and SSR5 NA indicated that this variety was not included in the genetical studies from where assignations were derived.

| **Use** |  |  |  |  |  |
| --- | --- | --- | --- | --- | --- |
| Wine | Table | Mixed |  |  |  |
| 8907 (295) | 3106 (104) | 1676 (56) |  |  |  |
|  |  |  |  |  |  |
| **Geo** |  |  |  |  |  |
| EUR_WEST | BALKANS | MED_EAST | EUR_IBER | EUR_ITAL | NA |
| 3120 (104) | 3120 (103) | 2757 (92) | 1500 (49) | 1439 (48) | 1456 (49) |
|  |  |  |  |  |  |
| **Geo × Use** |  |  |  |  |  |
| EUR_WEST_Wine | BALKANS_Wine | MED_EAST_Wine | EUR_ITAL_Wine | EUR_IBER_Wine | MED_EAST_Table |
| 2700 (90) | 2100 (69) | 1587 (53) | 1080 (36) | 990 (33) | 930 (31) |
| BALKANS_Table | EUR_IBER_Table | EUR_ITAL_Table | EUR_WEST_Table | | NA |
| 570 (19) | 210 (7) | 149 (5) | 120 (4) |  | 3553 (118) |
|  |  |  |  |  |  |
|  |  |  |  |  |  |
| **SNP4** |  |  |  |  |  |
| WEST_Wine | EAST_Table | BALK_Wine | IBER_Wine | Admixture/NA |  |
| 930 (31) | 717 (24) | 570 (18) | 360 (12) | 5990 (199) |  |
|  |  |  |  |  |  |
| **SSR5** |  |  |  |  |  |
| WCEUR_Wine | BALK_Wine | EAST_Table | IBER_WT | ITACE_Table | Admixture/NA |
| (1140) 38 | 930 (30) | 713 (24) | 420 (14) | 120 (4) | 4977 (166) |

**Table B ESM.** Summary of sample sizes for each cofactor and each class in terms of number of pips. Number of varieties is presented between brackets.

|  | **threshold** | **median** | |  | **min** | | |  | **max** | | |
| --- | --- | --- | --- | --- | --- | --- | --- | --- | --- | --- | --- |
|  |  | acc (%) | filt (%) |  | acc (%) | filt (%) | corresp. class |  | acc (%) | filt (%) | corresp. class |
|  |  |  |  |  |  |  |  |  |  |  |  |
|  | 0 | 80 | 0 |  | 79 | 0 | Table |  | 80 | 0 | Wine |
| **Use** | 0.5 | 80 | 0 |  | 79 | 0 | Table |  | 80 | 0 | Wine |
|  | 0.8 | 91 | 46 |  | 91 | 47 | Table |  | 91 | 45 | Wine |
|  |  |  |  |  |  |  |  |  |  |  |  |
|  | 0 | 42 | 0 |  | 36 | 0 | EUR_WEST |  | 50 | 0 | EUR_IBER |
| **Geo** | 0.5 | 55 | 76 |  | 48 | 77 | EUR_ITAL |  | 76 | 77 | EUR_IBER |
|  | 0.8 | 86 | 99 |  | 38 | 99 | BALKANS |  | 92 | 99 | EUR_IBER |
|  |  |  |  |  |  |  |  |  |  |  |  |
|  | 0 | 33 | 0 |  | 29 | 0 | BALKANS_Wine |  | 69 | 0 | EUR_IBER_Table |
| **Geo × Use** | 0.5 | 44 | 64 |  | 28 | 69 | BALKANS_Wine |  | 85 | 50 | EUR_WEST_Table |
|  | 0.8 | 50 | 94 |  | 17 | 96 | BALKANS_Wine |  | 100 | 82 | EUR_WEST_Table |
|  |  |  |  |  |  |  |  |  |  |  |  |
|  | 0 | 73 | 0 |  | 66 | 0 | BALK_Wine |  | 82 | 0 | EAST_Table |
| **SNP4** | 0.5 | 78 | 13 |  | 69 | 13 | BALK_Wine |  | 87 | 9 | EAST_Table |
|  | 0.8 | 89 | 58 |  | 84 | 61 | BALK_Wine |  | 97 | 33 | EAST_Table |
|  |  |  |  |  |  |  |  |  |  |  |  |
|  | 0 | 60 | 0 |  | 52 | 0 | ITACE_Table |  | 63 | 0 | EAST_Table |
| **SSR** | 0.5 | 68 |  |  | 59 | 30 | ITACE_Table |  | 73 | 23 | EAST_Table |
|  | 0.8 | 82 | 78 |  | 70 | 82 | ITACE_Table |  | 90 | 61 | EAST_Table |

**Table C ESM**: Summary of class accuracies according to posterior probability cut-off. Results are presented for each covariate of interest included here, and for three different filtering threshold values: 0 (no filtering), 0.5 and 0.8. The median, minimum and maximum class accuracies values are presented (acc), next to the corresponding proportion of pips filtered out (filt). For extrema values, the corresponding class is reported.

|  | **threshold** | **median** | |  | **min** | | |  | **max** | | |
| --- | --- | --- | --- | --- | --- | --- | --- | --- | --- | --- | --- |
|  |  | acc (%) | filt (%) |  | acc (%) | filt (%) | corresp. class |  | acc (%) | filt (%) | corresp. class |
|  |  |  |  |  |  |  |  |  |  |  |  |
|  | 0 | 80 | 0 |  | 79 | 0 | Table |  | 80 | 0 | WINE |
| **Use** | 0.5 | 80 | 0 |  | 79 | 0 | Table |  | 80 | 0 | WINE |
|  | 0.8 | 81 | 3 |  | 80 | 3 | Table |  | 81 | 3 | WINE |
|  |  |  |  |  |  |  |  |  |  |  |  |
|  | 0 | 43 | 0 |  | 37 | 0 | EUR_WEST |  | 51 | 0 | EUR_IBER |
| **Geo** | 0.5 | 43 | 1 |  | 37 | 1 | EUR_WEST |  | 51 | 1 | EUR_IBER |
|  | 0.8 | 44 | 15 |  | 38 | 17 | EUR_WEST |  | 55 | 14 | EUR_IBER |
|  |  |  |  |  |  |  |  |  |  |  |  |
|  | 0 | 39 | 0 |  | 31 | 0 | BALKANS_Wine |  | 80 | 0 | EUR_IBER_Table |
| **Geo × Use** | 0.5 | 41 | 18 |  | 31 | 22 | BALKANS_Wine |  | 82 | 7 | EUR_IBER_Table |
|  | 0.8 | 45 | 62 |  | 33 | 69 | BALKANS_Wine |  | 91 | 32 | EUR_WEST_Table |
|  |  |  |  |  |  |  |  |  |  |  |  |
|  | 0 | 76 | 0 |  | 69 | 0 | BALK_Wine |  | 83 | 0 | EAST_Table |
| **SNP4** | 0.5 | 76 | 0 |  | 70 | 0 | BALK_Wine |  | 83 | 0 | EAST_Table |
|  | 0.8 | 80 | 11 |  | 72 | 13 | BALK_Wine |  | 86 | 8 | EAST_Table |
|  |  |  |  |  |  |  |  |  |  |  |  |
|  | 0 | 60 | 0 |  | 52 | 0 | ITACE_Table |  | 63 | 0 | EAST_Table |
| **SSR5** | 0.5 | 68 | 29 |  | 59 | 30 | ITACE_Table |  | 73 | 23 | EAST_Table |
|  | 0.8 | 82 | 78 |  | 70 | 82 | ITACE_Table |  | 90 | 61 | EAST_Table |

**Table D ESM:** Summary of class accuracies according to cut-off based on the proportion of cases among 100 permutations where each pip was classified to a given group. Results are presented for each covariate of interest and for three different filtering threshold values: 0 (no filtering), 0.5 and 0.8. The median, minimum and maximum class accuracies values are presented (acc), next to the corresponding proportion of pips filtered out (filt). For extrema values, the corresponding class is reported.

**Figure legends**

**Figure A ESM**: Observed distributions of pip lengths for the three size classes of modern berry sizes and those observed for the two archaeological phases.

**Figure B ESM**: Principal Component Analysis calculated on Fourier coefficients of all modern pips. Each biplot is a subset for the covariate (column) x class (row) of interest, only showing concerned varieties as their average scores on PC1 and PC2. The red envelope is obtained with a kernel density estimate to visualize the relative positions of each class.

**Figure C ESM**: Unrooted tree obtained through hierarchical clustering of pip form (length + shape) grouped according to all geographical groups. For each node the numbers correspond to pvclust/cross-validation values.

**Figure D ESM**. Class accuracies (second row) for discriminant analyses. Boxplots display class accuracies obtained over 100 permutations of balanced datasets. Red lines indicate median (solid), 10% and 90% centiles (dashed), min and max values (dotted) obtained by chance alone among 100 permutations).

**Figure E ESM**: Sample size versus class accuracies. Rows correspond to filtering out individual predictions based on posterior probability (first row) or the number of occurrences among permutations (second row); columns correspond to each model presented in this study. For each panel, the x-axis represents the cut-off criterion, the y-axis represents both the class accuracies obtained (solid lines and confidence intervals) and the proportion of sample size retained (dashed lines).

**Figure F.** Inferences for archaeological pips of the domesticated type from the Mas de Vignoles XIV. The columns correspond to the different models presented after an inference on the wild/domesticated. The rows correspond to different filtering options: no filtering (which is Figure 3), filtering out pips with a posterior probability < 0.5, or those predicted less than 50% of the 100 permutations with the same label.
