# Supplementary figures and images for: Pip shape echoes grapevine domestication history
Source: Sci Rep. 2021 Nov 1;11:21381. doi: 10.1038/s41598-021-00877-4 (PMC8560759; doi:10.1038/s41598-021-00877-4)

Modern pips

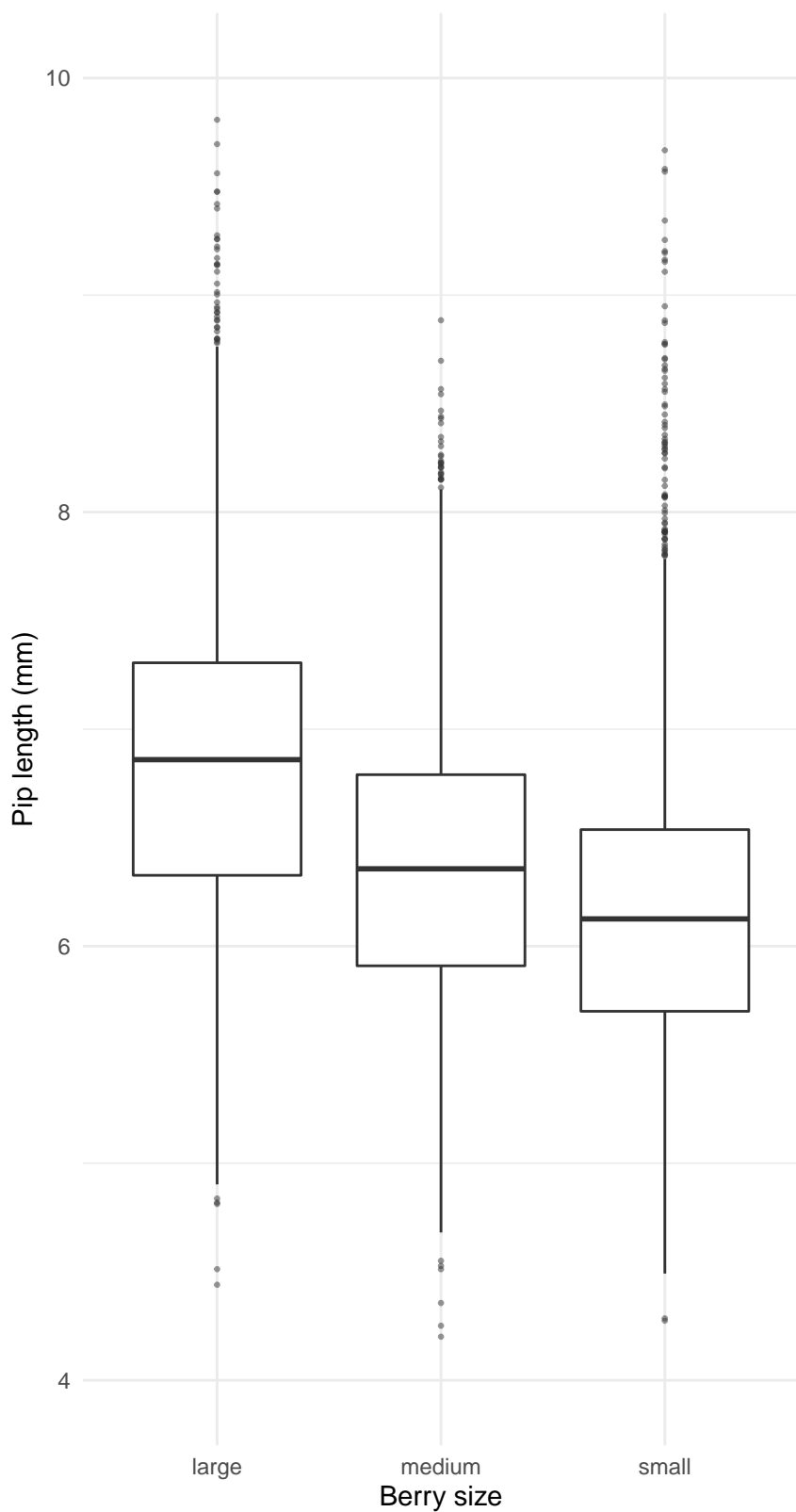

Archaeological pips

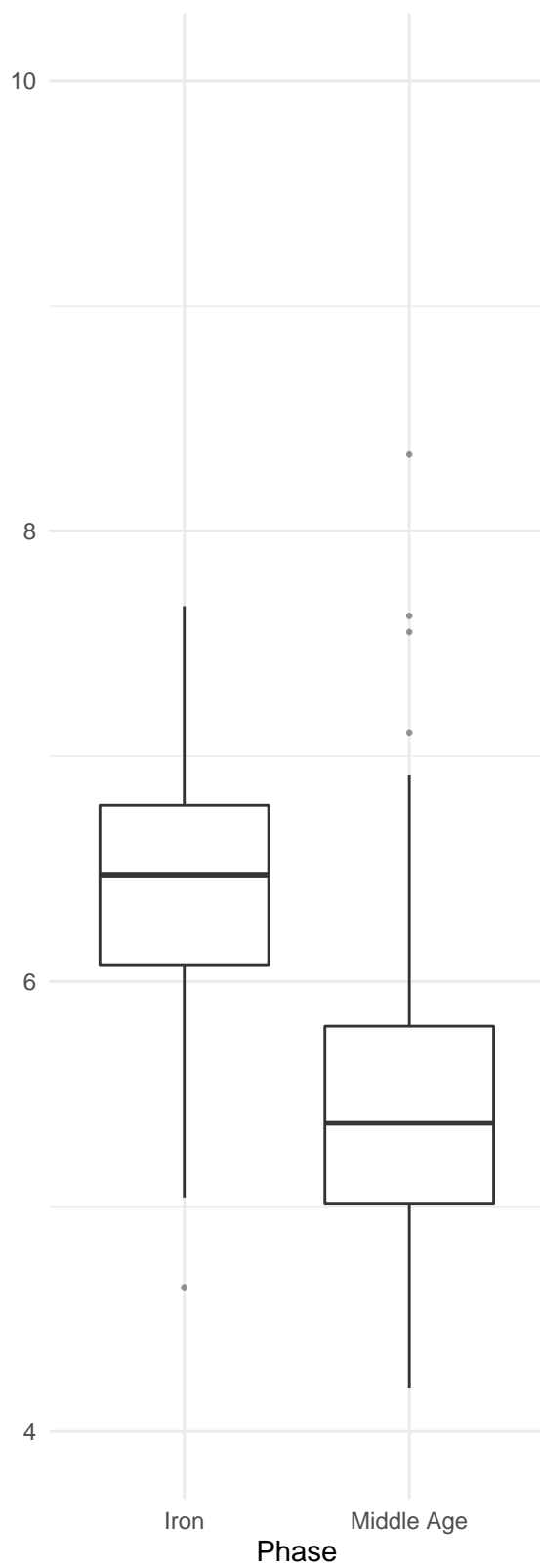

Supplement: Supplementary file 2 — Supplementary Information 2. [file 41598_2021_877_MOESM2_ESM.pdf]

a) Usage

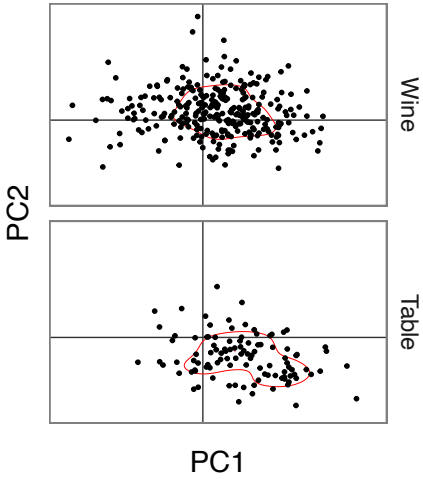

b) Geo (core)

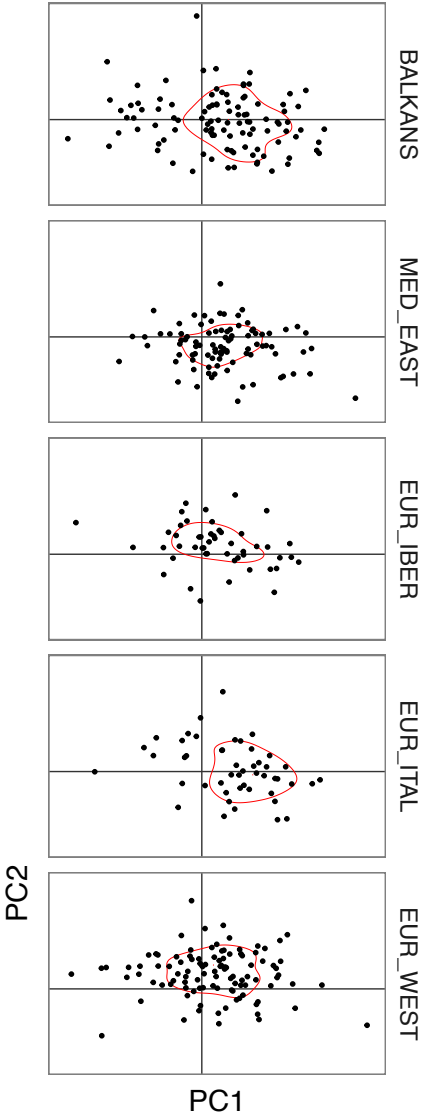

c) Geo (core) + usage

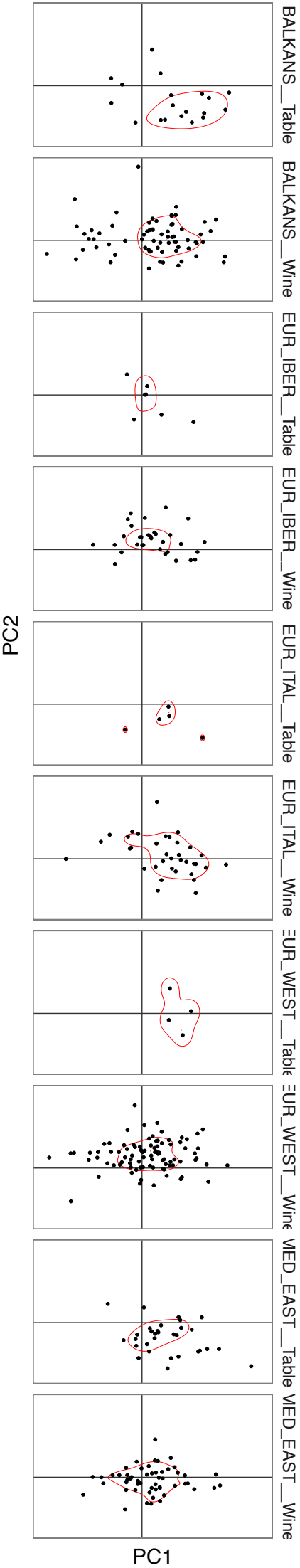

d) SNP4

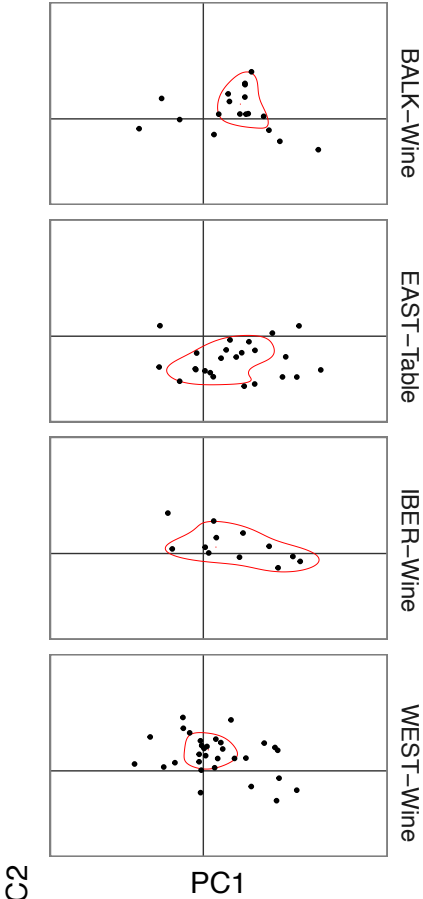

e) SSR5

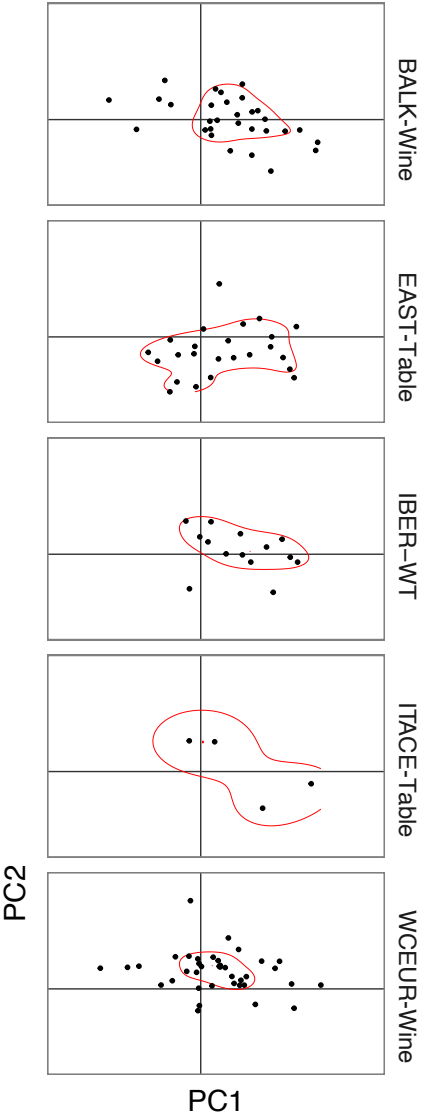

Supplement: Supplementary file 3 — Supplementary Information 3. [file 41598_2021_877_MOESM3_ESM.pdf]

# Geo (full)

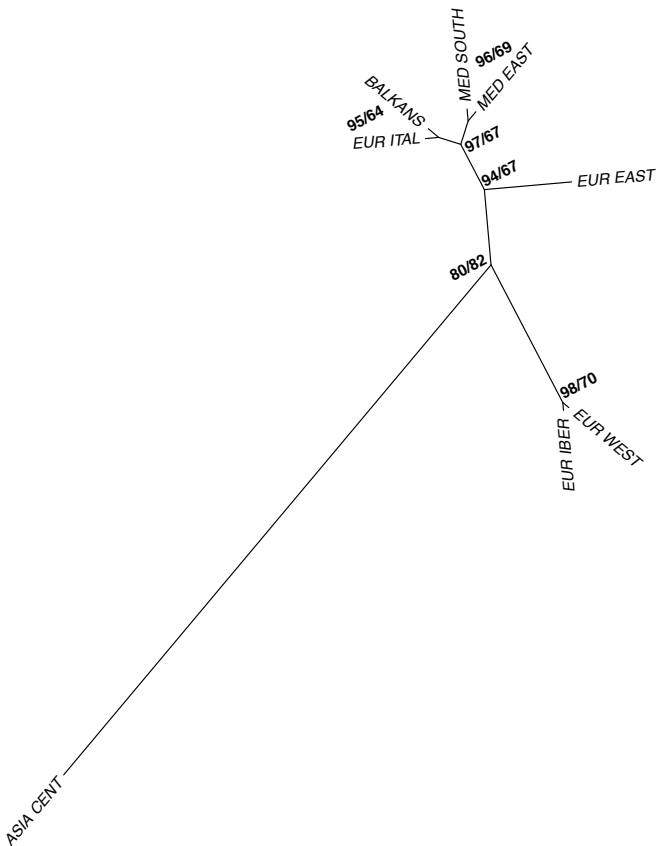

Supplement: Supplementary file 4 — Supplementary Information 4. [file 41598_2021_877_MOESM4_ESM.pdf]

a) Usage

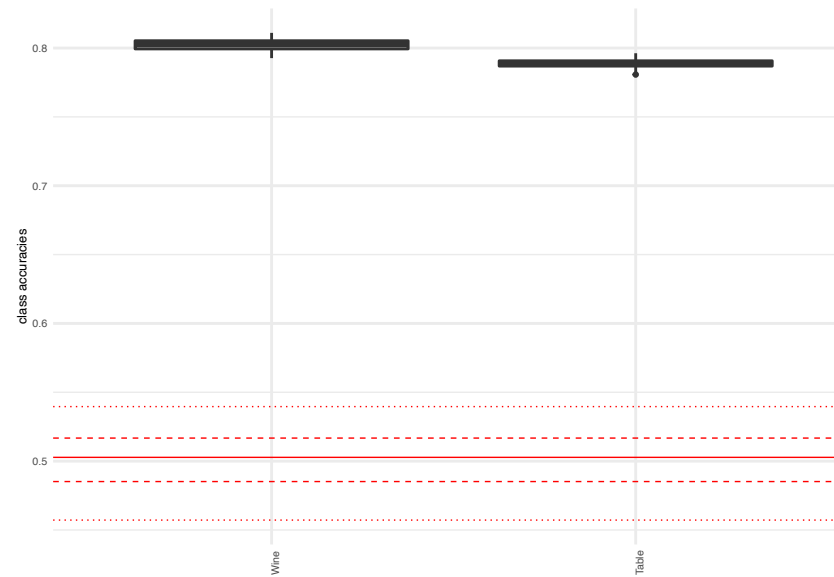

b) Geo

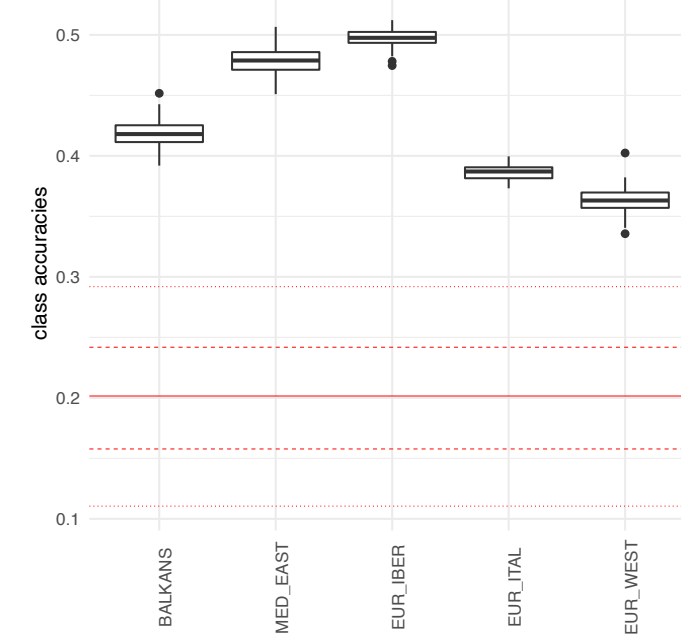

c) Geo × Usage

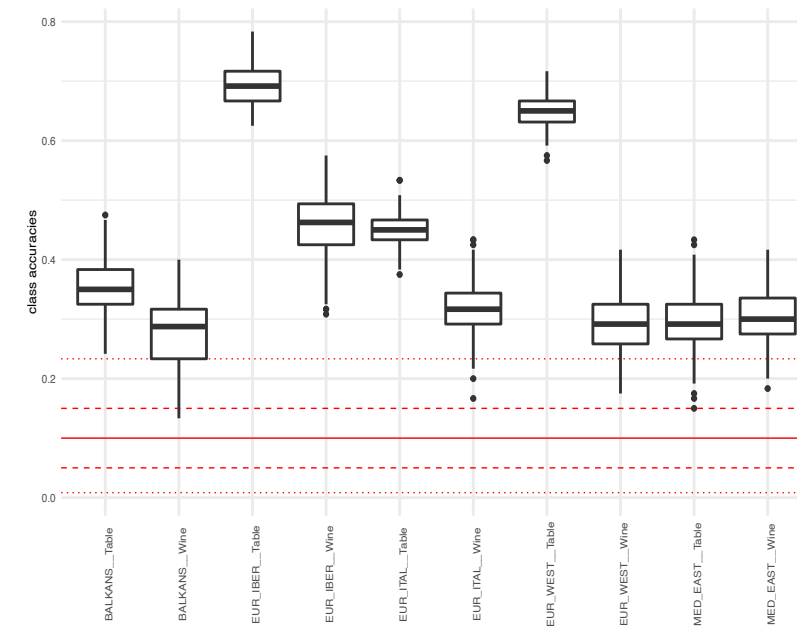

d) SNP4

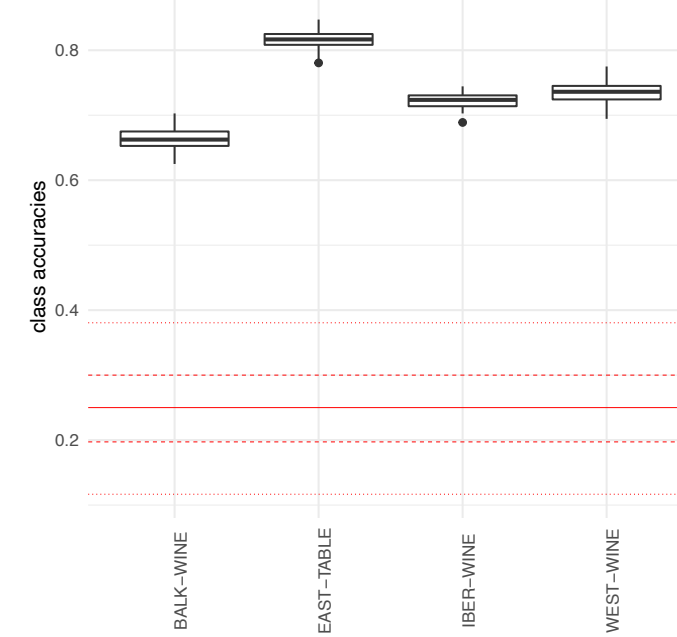

e) SSR5

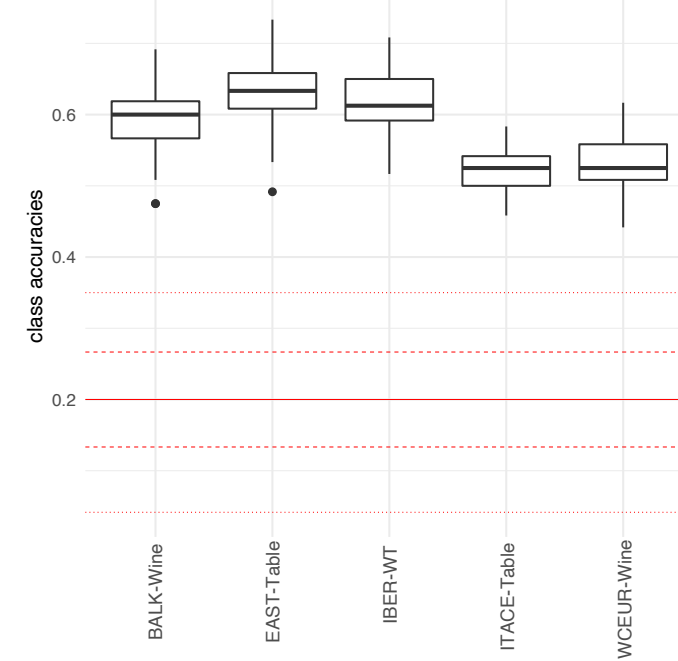

Supplement: Supplementary file 5 — Supplementary Information 5. [file 41598_2021_877_MOESM5_ESM.pdf]

no filtering

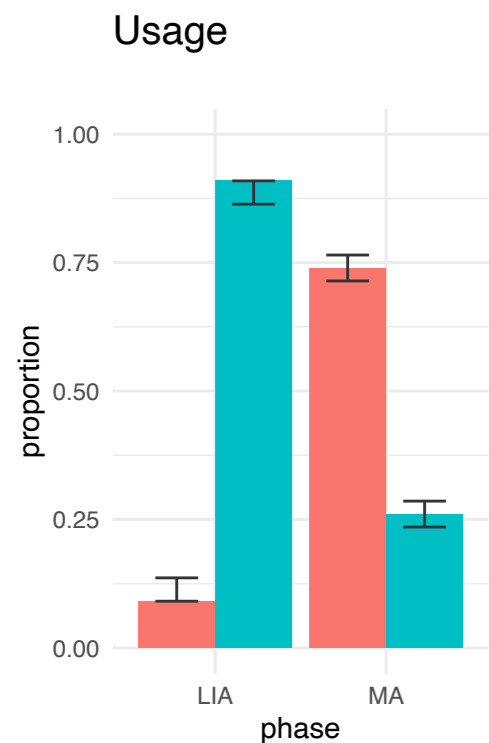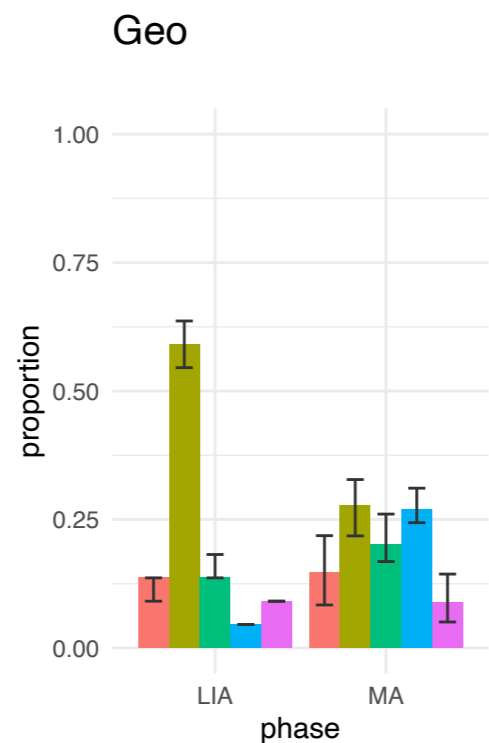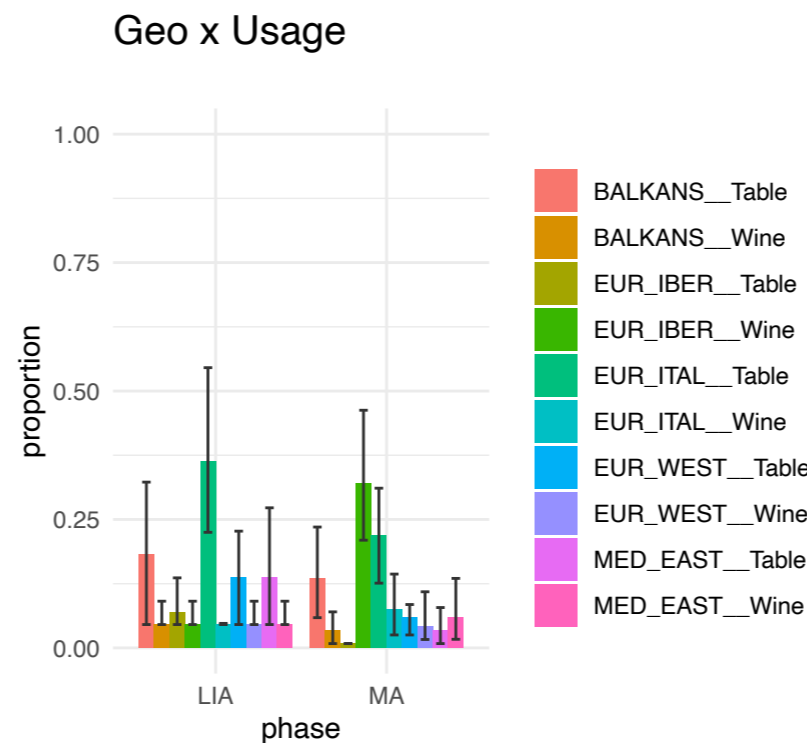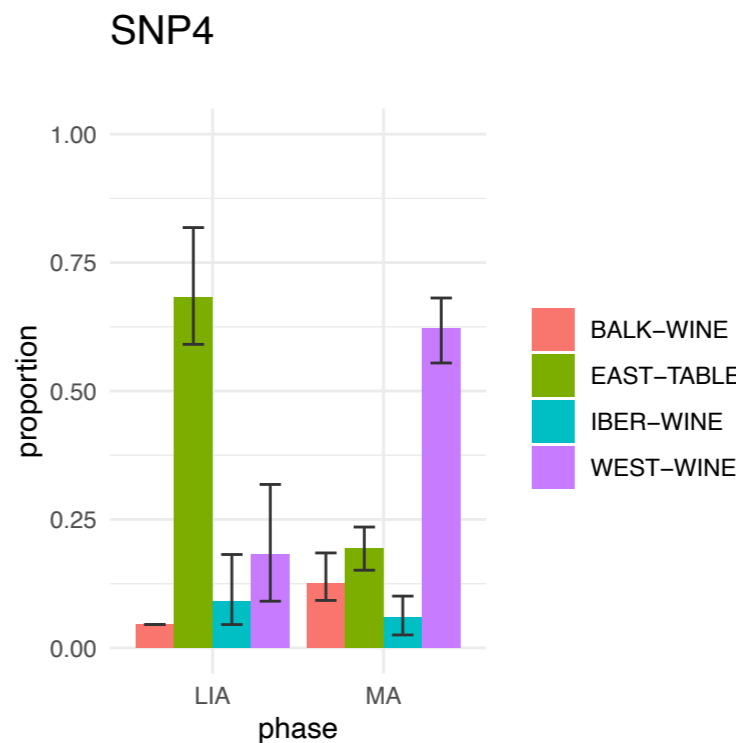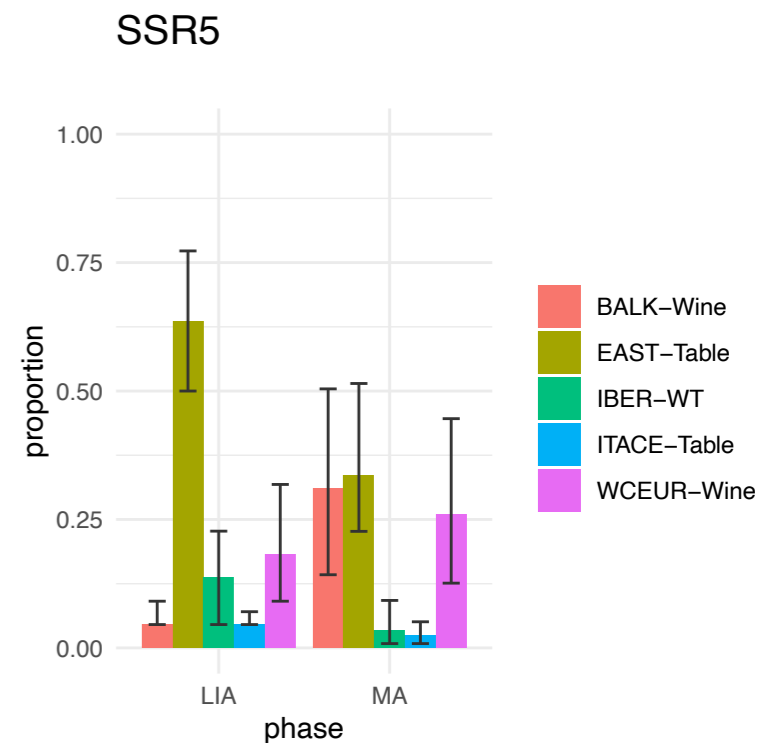

posterior  $\geq 0.5$

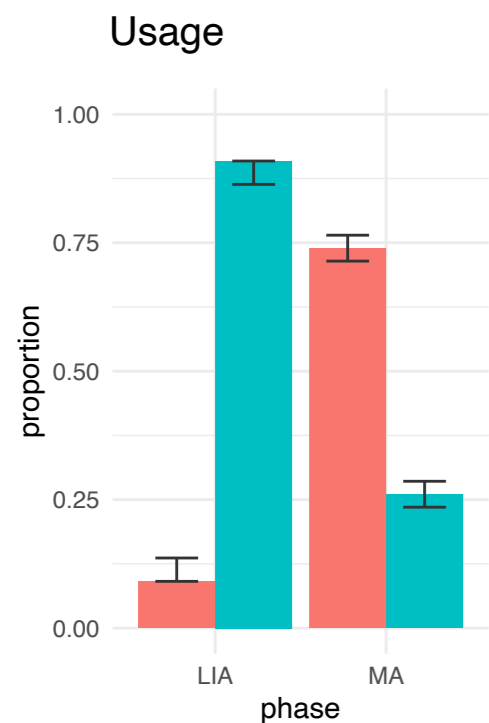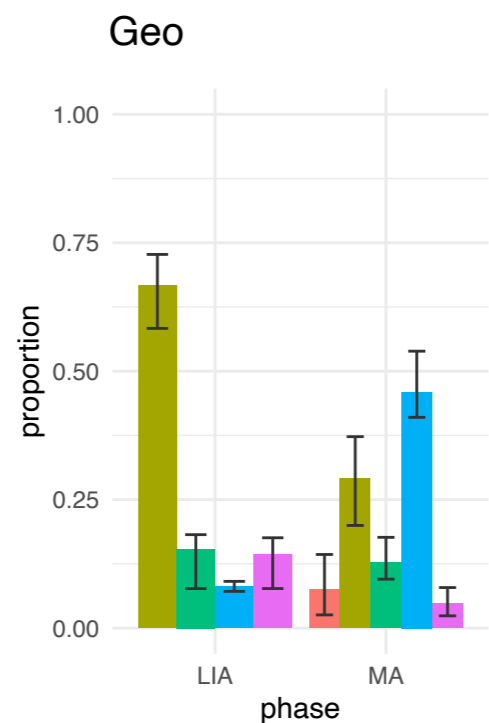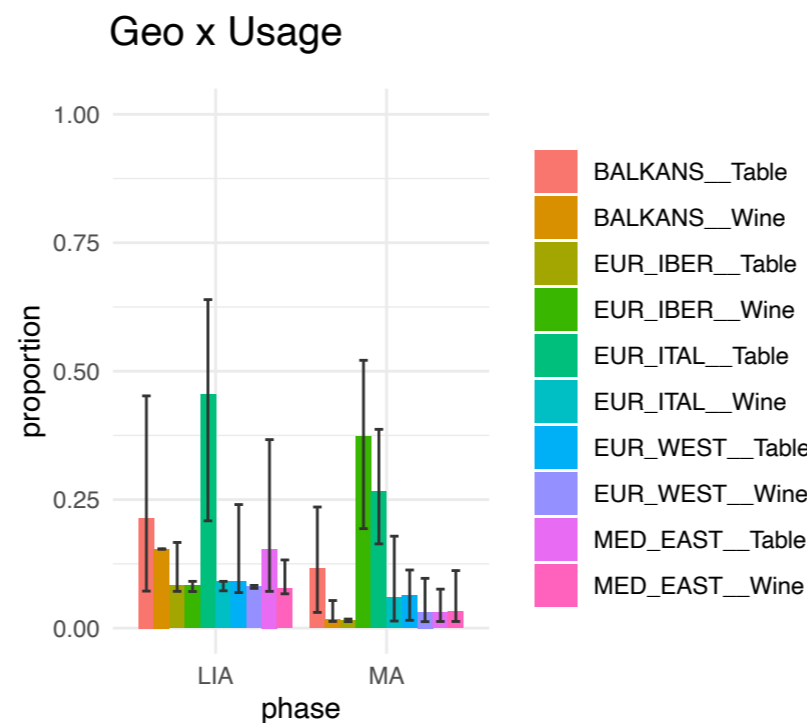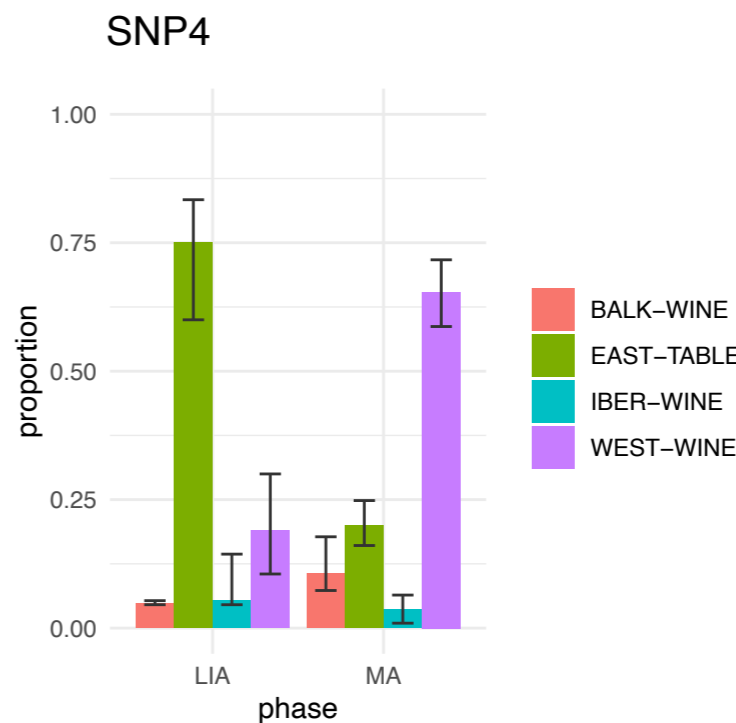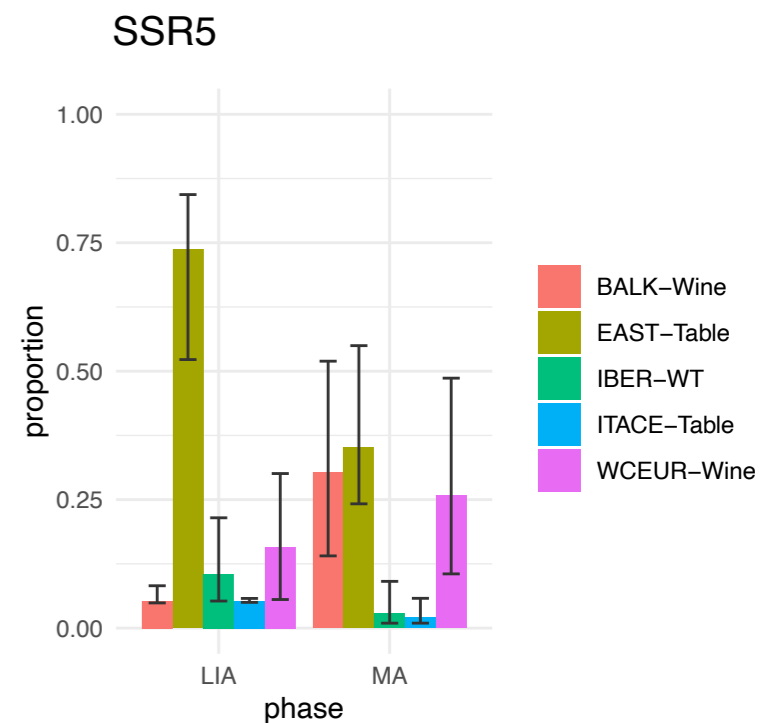

counting  $\geq 50\%$

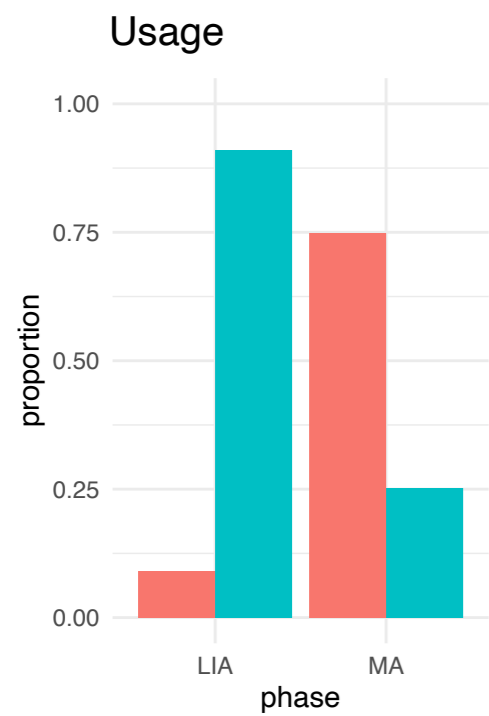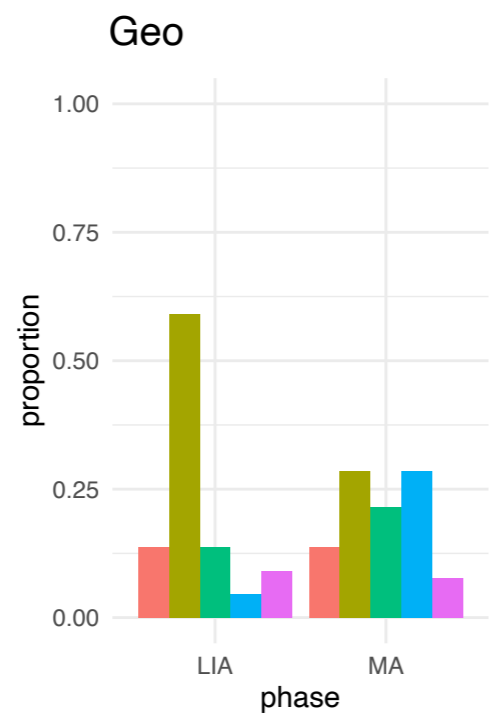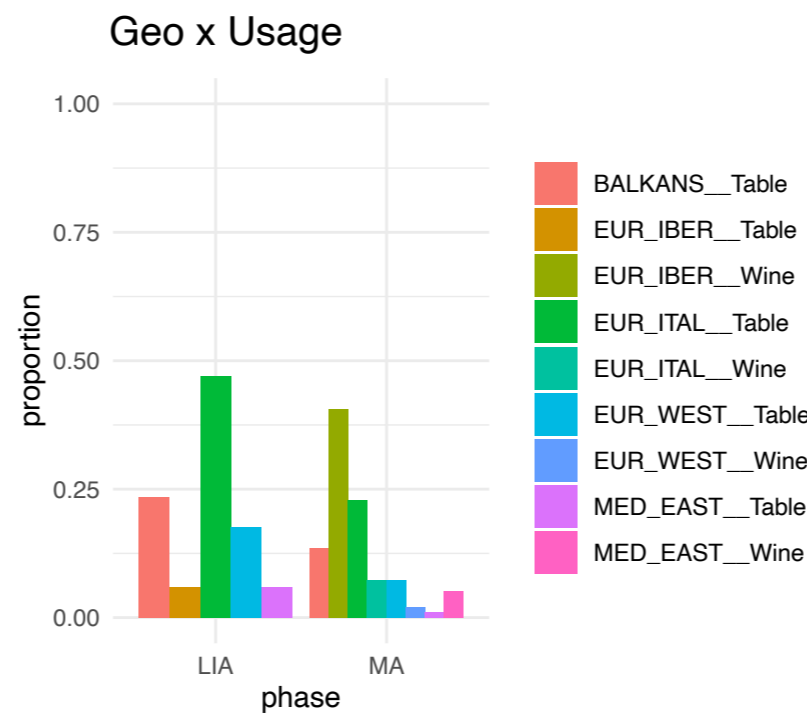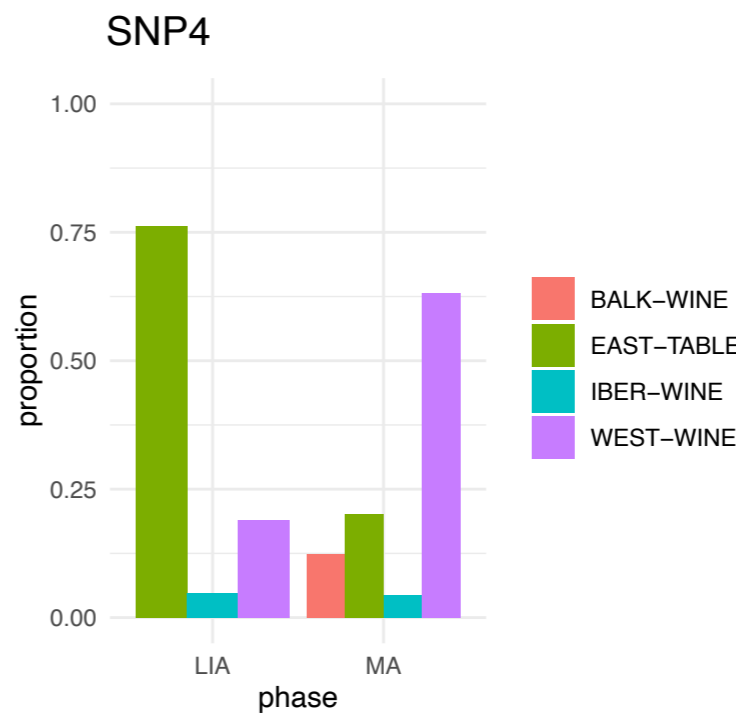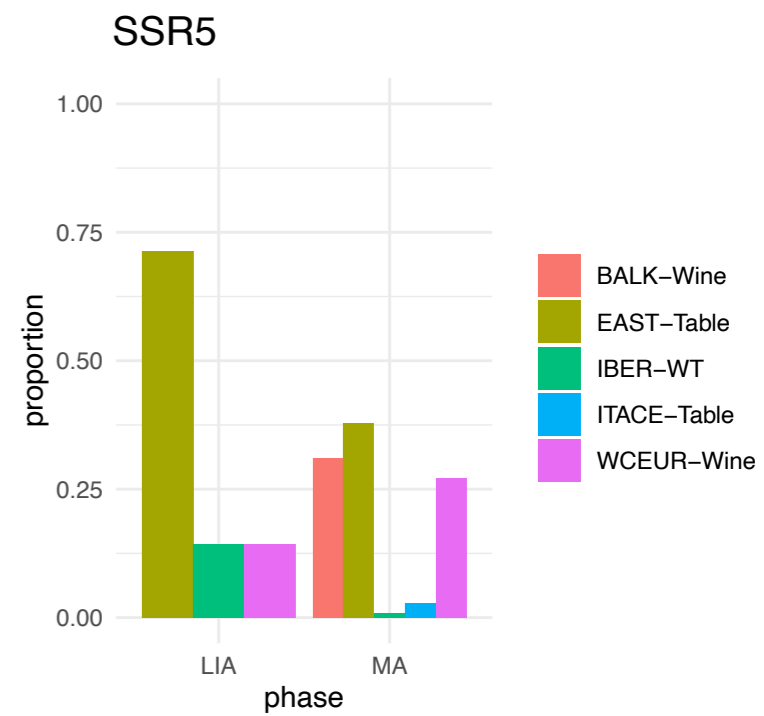

Supplement: Supplementary file 7 — Supplementary Information 7. [file 41598_2021_877_MOESM7_ESM.pdf]
