# Supplementary material for: Pip shape echoes grapevine domestication history
Source: Sci Rep. 2021 Nov 1;11:21381. doi: 10.1038/s41598-021-00877-4 (PMC8560759; doi:10.1038/s41598-021-00877-4)
Supplement: Supplementary file 6 — Supplementary Information 6. [file 41598_2021_877_MOESM6_ESM.pdf]

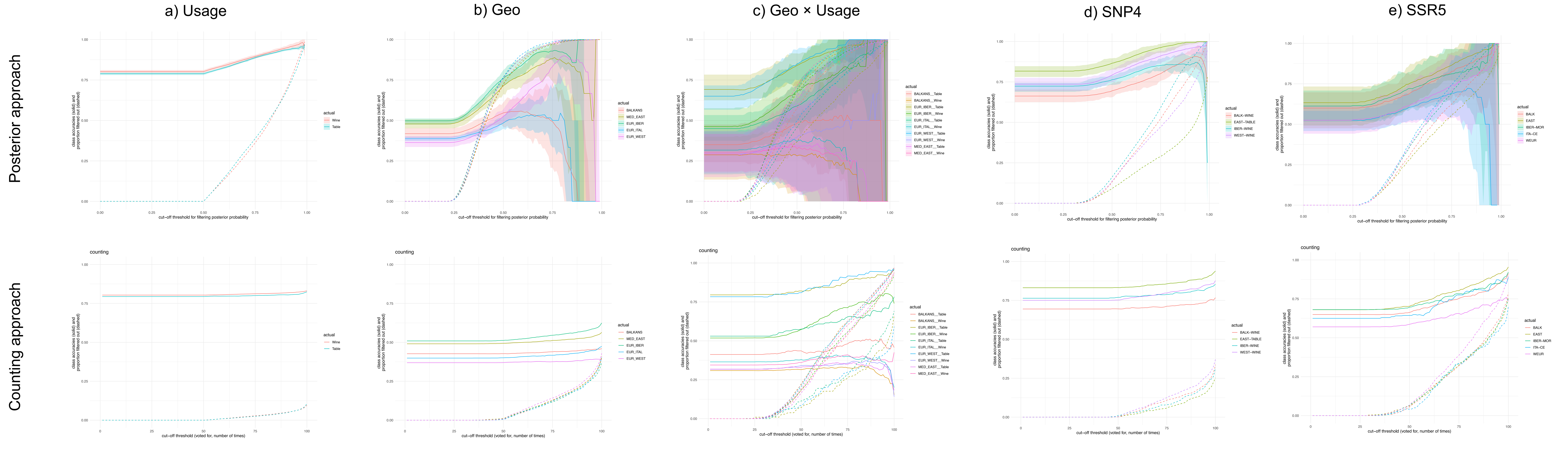
a) Usage
b) Geo
c) Geo × Usage
d) SNP4
e) SSR5
Posterior approach
Counting approach
class accuracies (solid) and proportion filtered out (dashed)
cut−off threshold for filtering posterior probability
cut−off threshold (voted for, number of times)
counting
actual
Wine
Table
BALKANS
MED_EAST
EUR_IBER
EUR_ITAL
EUR_WEST
BALKANS__Table
BALKANS__Wine
EUR_IBER__Table
EUR_IBER__Wine
EUR_ITAL__Table
EUR_ITAL__Wine
EUR_WEST__Table
EUR_WEST__Wine
MED_EAST__Table
MED_EAST__Wine
BALK−WINE
EAST−TABLE
IBER−WINE
WEST−WINE
BALK
EAST
IBER−MOR
ITA−CE
WEUR
