# Supplementary material for: Pip shape echoes grapevine domestication history
Source: Sci Rep. 2021 Nov 1;11:21381. doi: 10.1038/s41598-021-00877-4 (PMC8560759; doi:10.1038/s41598-021-00877-4)
Supplement: Supplementary file 8 — Supplementary Information 8. [file 41598_2021_877_MOESM8_ESM.pdf]

Variety	Pip length (mm)	Usage	Origin	SNP4	SSR5	N
Abjouch	8.78 ± 0.507	Table	ASIA_CENT	NA	EAST_Table	30
Abouhou	7.19 ± 0.313	Table	MED_SOUTH	EAST_Table	Admixture	30
Adanasuri	7.16 ± 0.38	Wine	MED_EAST	NA	NA	30
Adreuli tetri	6.78 ± 0.312	Table	MED_EAST	NA	NA	30
Adzniji	7.12 ± 0.36	Table	MED_EAST	NA	NA	30
Aetonychi kokkino	7.28 ± 0.473	Table	BALKANS	NA	NA	30
Aetonychi lefko	6.76 ± 0.298	Table	BALKANS	NA	Admixture	30
Ag isioum	5.65 ± 0.286	Wine	MED_EAST	Admixture	NA	30
Agadaï	6.98 ± 0.263	Table	EUR_EAST	Admixture	EAST_Table	30
Agiorgitiko	6.8 ± 0.23	Wine	BALKANS	NA	NA	30
Aglianico	6.51 ± 0.221	Wine	EUR_ITAL	NA	Admixture	30
Agoumastos	5.89 ± 0.206	Wine	NA	NA	NA	30
Ahmeur bou Ahmeur	6.89 ± 0.327	Table	MED_SOUTH	Admixture	NA	30
Aïdani mavro	6.56 ± 0.281	Wine	BALKANS	Admixture	Admixture	30
Aïn el Bouma	5.59 ± 0.376	Table	MED_SOUTH	Admixture	NA	30
Airén	6.82 ± 0.212	Mixed	EUR_IBER	IBER_Wine	IBER_WT	30
Aladasturi	7.05 ± 0.274	Wine	MED_EAST	NA	NA	30
Alarije	5.73 ± 0.297	Wine	EUR_IBER	IBER_Wine	NA	30
Albaranzeuli bianco	6.38 ± 0.377	Wine	EUR_ITAL	Admixture	Admixture	30
Alexandroouli	6.57 ± 0.231	Wine	MED_EAST	Admixture	NA	30
Alfrocheiro preto	5.52 ± 0.219	Wine	EUR_IBER	WEST_Wine	NA	30
Aligoté	6.23 ± 0.322	Wine	EUR_WEST	NA	WCEUR_Wine	30
Alvarinho	5.24 ± 0.256	Wine	EUR_IBER	WEST_Wine	Admixture	30
Amaral	5.88 ± 0.201	Wine	EUR_IBER	WEST_Wine	WCEUR_Wine	30
Amessasse	6.54 ± 0.305	Table	MED_SOUTH	NA	NA	30
Amigne	5.22 ± 0.27	Wine	EUR_WEST	Admixture	Admixture	30
Amokrane	7.06 ± 0.429	Table	MED_SOUTH	NA	Admixture	27
Ancellotta	5.85 ± 0.18	Wine	EUR_ITAL	WEST_Wine	Admixture	30
Ansonica	6.9 ± 0.27	Mixed	EUR_ITAL	Admixture	Admixture	30
Araklinos	6.55 ± 0.347	Wine	BALKANS	Admixture	Admixture	30
Aramon	6.05 ± 0.351	Wine	EUR_WEST	NA	NA	30
Arbane	6.91 ± 0.386	Wine	EUR_WEST	Admixture	Admixture	30
Arinto do Dão	5.85 ± 0.453	Mixed	EUR_IBER	NA	NA	60
Arna−guirna	7.53 ± 0.181	Wine	MED_EAST	Admixture	Admixture	30
Arneis	6.74 ± 0.235	Wine	EUR_ITAL	Admixture	Admixture	30
Arrouya	6.19 ± 0.321	Wine	EUR_WEST	WEST_Wine	Admixture	30
Arvine	5.71 ± 0.34	Wine	EUR_WEST	WEST_Wine	Admixture	30
Asprouda Patron	6.54 ± 0.307	Wine	BALKANS	NA	Admixture	30
Asprouda Santorinis	6.59 ± 0.21	Wine	BALKANS	Admixture	Admixture	30
Assali al arous	7.55 ± 0.421	Table	MED_EAST	EAST_Table	EAST_Table	30
Assouad karech	6.54 ± 0.291	Wine	MED_EAST	EAST_Table	EAST_Table	30
Assouad koupti	6.3 ± 0.315	Wine	MED_EAST	Admixture	EAST_Table	30
Asswad Abou Khislé	8.45 ± 0.482	Table	MED_EAST	EAST_Table	EAST_Table	30
Assyrtiko	6.91 ± 0.362	Wine	BALKANS	Admixture	NA	30
Asuretuli shavi	6.08 ± 0.26	Wine	MED_EAST	NA	NA	30
Athiri (Thrapsathiri)	6.13 ± 0.332	Mixed	BALKANS	NA	Admixture	30
Avassirkhva	6.49 ± 0.271	Wine	MED_EAST	Admixture	Admixture	30
Babeasca neagra	5.57 ± 0.264	Wine	BALKANS	NA	NA	30
Babica crna	6.21 ± 0.319	Wine	BALKANS	Admixture	Admixture	30
Bachet	6.55 ± 0.363	Wine	EUR_WEST	NA	Admixture	30
Badagui noir	6.53 ± 0.223	Wine	MED_EAST	Admixture	Admixture	30
Bajor kék	7.58 ± 0.309	Wine	BALKANS	BALK_Wine	NA	30
Bakhtiori	6.84 ± 0.404	Wine	ASIA_CENT	Admixture	NA	30
Baratcsuha szürke	6.27 ± 0.319	Wine	BALKANS	Admixture	Admixture	30
Barbera	6.99 ± 0.375	Wine	EUR_ITAL	NA	Admixture	30
Baresana	7.4 ± 0.327	Table	EUR_ITAL	EAST_Table	NA	30
Baserri	5.91 ± 0.232	Wine	EUR_WEST	WEST_Wine	WCEUR_Wine	30
Basicata	5.59 ± 0.315	Table	BALKANS	BALK_Wine	BALK_Wine	60
Batoca	6.26 ± 0.215	Wine	EUR_IBER	NA	NA	30
Batuta neagra	6.48 ± 0.306	Mixed	BALKANS	NA	BALK_Wine	30
Bayadi de Syrie	6.59 ± 0.355	Table	MED_EAST	Admixture	Admixture	30
Bayadi du Liban	7.24 ± 0.412	Table	MED_EAST	NA	EAST_Table	30
Bayan shirei	6.28 ± 0.316	Wine	MED_EAST	Admixture	EAST_Table	30
Bellone	7.14 ± 0.275	Wine	EUR_ITAL	Admixture	Admixture	30
Benedicto de Aragon	5.98 ± 0.364	NA	NA	NA	NA	30
Berbecel	5.9 ± 0.337	Wine	BALKANS	NA	BALK_Wine	30
Bermestia bianca	7.54 ± 0.289	Table	EUR_ITAL	Admixture	Admixture	30
Beylerce	5.85 ± 0.271	Mixed	MED_EAST	NA	Admixture	30
Bicane	7.79 ± 0.318	Table	NA	Admixture	Admixture	30
Black morocco	8.8 ± 0.461	Table	MED_SOUTH	Admixture	NA	30
Bobal	5.74 ± 0.304	Wine	EUR_IBER	Admixture	NA	30
Bondola	6.83 ± 0.304	Wine	EUR_WEST	Admixture	Admixture	30
Borchalo	6.16 ± 0.239	Wine	MED_EAST	NA	NA	30
Bourboulenc	6.81 ± 0.301	Wine	EUR_WEST	Admixture	NA	30
Bouteillan noir	6.28 ± 0.318	Wine	EUR_WEST	NA	NA	30
Brachetto	5.35 ± 0.369	Wine	EUR_ITAL	Admixture	Admixture	30
Braghina	6.8 ± 0.272	Mixed	BALKANS	BALK_Wine	NA	30
Brun fourca	7.29 ± 0.443	Wine	EUR_WEST	Admixture	NA	30
Bzvanura	5.38 ± 0.346	Wine	MED_EAST	NA	NA	30
Cabernet franc	7.8 ± 0.463	Wine	EUR_WEST	Admixture	WCEUR_Wine	30
Cabernet−Sauvignon	5.29 ± 0.507	Wine	EUR_WEST	NA	NA	30
Cahours	7.01 ± 0.242	Wine	EUR_WEST	Admixture	Admixture	30
Caino bravo	6.31 ± 0.25	NA	NA	NA	NA	30
Calabrese	6.72 ± 0.32	Wine	EUR_ITAL	Admixture	NA	30
Calitor blanc de semis	6.37 ± 0.334	Wine	EUR_WEST	NA	NA	30
Carcajolo	6.94 ± 0.416	Wine	EUR_IBER	Admixture	NA	30
Cardinal	6.43 ± 0.467	Table	NEW_WORLD	Admixture	NA	30
Carignan	5.78 ± 0.505	Wine	EUR_IBER	Admixture	NA	30
Casetta	7.14 ± 0.24	Wine	EUR_ITAL	WEST_Wine	Admixture	30
Castelão	6.17 ± 0.341	Mixed	EUR_IBER	Admixture	NA	30
Castellana blanca	6.53 ± 0.187	Wine	EUR_IBER	Admixture	NA	30
Cayetana blanca	6.7 ± 0.288	Wine	EUR_IBER	IBER_Wine	NA	30
Chaani biely	6.69 ± 0.47	Table	MED_EAST	NA	NA	30
Chahnani	7.12 ± 0.238	Table	MED_EAST	Admixture	Admixture	30
Chami	6.91 ± 0.378	Table	MED_SOUTH	NA	Admixture	30
Chaouch blanc	7.03 ± 0.386	Table	MED_EAST	Admixture	NA	30
Chaptal	7.4 ± 0.533	Table	EUR_WEST	EAST_Table	Admixture	30
Chardonnay	5.17 ± 0.293	Wine	EUR_WEST	Admixture	WCEUR_Wine	30
Chasselas	6.56 ± 0.287	Mixed	EUR_WEST	WEST_Wine	NA	30
Chatus	5.33 ± 0.228	Wine	EUR_WEST	WEST_Wine	NA	30
Chekobali	6.74 ± 0.119	Wine	MED_EAST	NA	NA	30
Chevka	6.04 ± 0.36	Wine	BALKANS	NA	EAST_Table	30
Chinuri	6.85 ± 0.297	Wine	MED_EAST	NA	NA	30
Chiroka Melnichka	6.68 ± 0.251	Wine	BALKANS	NA	BALK_Wine	30
Chkaveri	6.51 ± 0.238	Wine	MED_EAST	NA	NA	30
Chouchillon	6.8 ± 0.297	Wine	EUR_WEST	WEST_Wine	WCEUR_Wine	30
Ciklap	6.32 ± 0.218	NA	NA	NA	NA	30
Cinsaut	6.31 ± 0.228	Mixed	EUR_WEST	Admixture	Admixture	30
Clairette	6.47 ± 0.44	Mixed	EUR_WEST	Admixture	NA	30
Coada vulpii	7.2 ± 0.181	Table	BALKANS	EAST_Table	Admixture	30
Coda di volpe bianca	5.75 ± 0.232	Wine	EUR_ITAL	Admixture	NA	30
Colorino	6.21 ± 0.324	Wine	EUR_ITAL	Admixture	Admixture	30
Cot	6.13 ± 0.456	Wine	EUR_WEST	NA	NA	30
Courbu	6.27 ± 0.268	Wine	EUR_WEST	WEST_Wine	NA	30
Criolla grande sanjuanina	6.77 ± 0.234	Mixed	NEW_WORLD	Admixture	ITACE_Table	30
Croatina	6.44 ± 0.345	Wine	EUR_ITAL	Admixture	NA	30
Dabouki	7.44 ± 0.4	Table	MED_EAST	EAST_Table	NA	30
Darkaia noir = Coarna neagra	6.36 ± 0.443	Table	MED_SOUTH	Admixture	NA	30
Dattier de Beyrouth	7.48 ± 0.376	Table	MED_EAST	Admixture	NA	30
Debina	7.2 ± 0.344	Wine	BALKANS	NA	BALK_Wine	30
Deckrot	5.22 ± 0.205	Wine	EUR_WEST	WEST_Wine	WCEUR_Wine	30
Dedo de Dama	7.79 ± 0.364	Table	EUR_IBER	Admixture	IBER_WT	30
Dermatas	7.54 ± 0.376	Mixed	BALKANS	Admixture	IBER_WT	30
Diminitis	6.78 ± 0.335	Wine	BALKANS	NA	NA	30
Dimrit	6.14 ± 0.378	Wine	MED_EAST	Admixture	Admixture	30
Dobricic	6.19 ± 0.24	Wine	BALKANS	NA	NA	30
Doight du neigne	7.21 ± 0.316	Table	MED_EAST	EAST_Table	Admixture	30
Dolcetto	5.91 ± 0.206	Wine	EUR_ITAL	Admixture	Admixture	30
Dominga	7.11 ± 0.393	Table	EUR_IBER	Admixture	Admixture	30
Donzelinho branco	5.97 ± 0.352	Wine	EUR_IBER	Admixture	NA	30
Dornefelder	6.58 ± 0.181	Wine	EUR_WEST	NA	Admixture	30
Doukkali = Bezoul et aouda ; Bezoul el Khadem	6.91 ± 0.345	Table	MED_SOUTH	NA	Admixture	30
Doux d'Henry	6.81 ± 0.273	Mixed	EUR_ITAL	NA	NA	30
Duras	5.7 ± 0.413	Wine	EUR_WEST	NA	WCEUR_Wine	30
Dureza	5.28 ± 0.284	Wine	EUR_WEST	WEST_Wine	WCEUR_Wine	30
Dziganidze	7.05 ± 0.281	Wine	MED_EAST	NA	NA	30
Endeladziseuli	6.55 ± 0.344	Wine	MED_EAST	NA	NA	30
Epinou	5.84 ± 0.233	Wine	EUR_WEST	Admixture	WCEUR_Wine	30
Espadeiro tinto	6.36 ± 0.237	Wine	EUR_IBER	Admixture	Admixture	30
Falanghina	6.7 ± 0.366	Wine	EUR_ITAL	Admixture	Admixture	30
Fer	6.47 ± 0.311	Wine	EUR_WEST	WEST_Wine	WCEUR_Wine	30
Ferlongo	6.74 ± 0.216	Table	EUR_IBER	Admixture	Admixture	30
Ferral tamara	6.77 ± 0.258	Table	EUR_IBER	NA	IBER_WT	30
Feruani	6.88 ± 0.291	Wine	MED_EAST	NA	NA	30
Feteasca alba	5.36 ± 0.23	Wine	BALKANS	Admixture	BALK_Wine	30
Fita alba	5.78 ± 0.213	Wine	BALKANS	NA	BALK_Wine	30
Flora	5.82 ± 0.213	Wine	NEW_WORLD	WEST_Wine	WCEUR_Wine	30
Fodja	8.26 ± 0.291	Table	MED_EAST	NA	NA	30
Fokiano	7.35 ± 0.251	Mixed	BALKANS	Admixture	Admixture	30
Fondo de Orza	6.32 ± 0.389	Table	EUR_IBER	Admixture	Admixture	30
François noir	6.35 ± 0.171	Wine	EUR_WEST	NA	WCEUR_Wine	30
Frankenthal rouge foncé	7.32 ± 0.245	Mixed	EUR_ITAL	NA	ITACE_Table	30
Fuella nera	6.32 ± 0.22	Wine	EUR_WEST	Admixture	Admixture	30
Furmint	7.17 ± 0.357	Wine	BALKANS	BALK_Wine	NA	30
Gaïdouria	5.58 ± 0.323	Wine	BALKANS	NA	Admixture	30
Galbena de Odobesti	7.48 ± 0.376	Wine	BALKANS	BALK_Wine	BALK_Wine	30
Galbena uriasa	6.84 ± 0.221	Wine	BALKANS	BALK_Wine	NA	30
Gamay	5.82 ± 0.367	Wine	EUR_WEST	Admixture	NA	30
Gantziandan	7.26 ± 0.316	Table	MED_EAST	Admixture	EAST_Table	30
Garganega	6.07 ± 0.265	Mixed	EUR_ITAL	Admixture	BALK_Wine	30
Garrido macho	7.09 ± 0.538	Mixed	EUR_IBER	IBER_Wine	IBER_WT	30
Gateta	5.27 ± 0.223	Mixed	EUR_IBER	Admixture	Admixture	30
Genk Uzum	6.87 ± 0.308	Table	MED_EAST	Admixture	Admixture	30
Genovèse	6.95 ± 0.331	Wine	EUR_WEST	Admixture	NA	30
Gibi	7.14 ± 0.421	Mixed	EUR_IBER	IBER_Wine	IBER_WT	30
Gines	6.01 ± 0.346	Table	NA	NA	NA	30
Glycostaphyllo	7.63 ± 0.323	Wine	MED_EAST	Admixture	Admixture	30
Glykerithra	5.94 ± 0.31	Wine	BALKANS	NA	Admixture	30
Golodan	7.3 ± 0.344	Table	ASIA_CENT	EAST_Table	EAST_Table	30
Gorula	6.5 ± 0.466	Table	MED_EAST	NA	NA	30
Goruli Mtsvane	6.99 ± 0.292	Wine	MED_EAST	NA	NA	30
Gouais blanc	5.09 ± 0.308	Wine	NA	BALK_Wine	NA	30
Grasa de Cotnari	7.42 ± 0.385	Wine	BALKANS	NA	BALK_Wine	30
Grec rouge	6.35 ± 0.232	Mixed	EUR_WEST	Admixture	Admixture	30
Grechetto	6.44 ± 0.304	Wine	EUR_ITAL	Admixture	Admixture	30
Greco bianco	6.81 ± 0.409	Wine	EUR_ITAL	Admixture	Admixture	30
Greco di Tufo	5.61 ± 0.253	Wine	EUR_ITAL	Admixture	Admixture	30
Grenache	5.39 ± 0.281	Wine	EUR_WEST	IBER_Wine	NA	30
Grenache blanc	5.36 ± 0.296	Wine	EUR_WEST	NA	NA	30
Grillo	6.11 ± 0.307	Wine	EUR_ITAL	Admixture	Admixture	30
Grk	7.03 ± 0.327	Wine	BALKANS	NA	Admixture	30
Gros Colman	6.92 ± 0.274	Table	EUR_EAST	Admixture	NA	30
Gros maroc	7.39 ± 0.329	Table	NA	Admixture	Admixture	30
Grüner Veltliner	7.27 ± 0.311	Wine	EUR_WEST	Admixture	Admixture	30
Hadari	6.75 ± 0.276	Table	MED_SOUTH	EAST_Table	Admixture	30
Haimka	6.65 ± 0.293	Mixed	BALKANS	NA	BALK_Wine	30
Hans	5.54 ± 0.232	Wine	EUR_WEST	NA	NA	30
Harslevelu	5.78 ± 0.422	Wine	BALKANS	BALK_Wine	BALK_Wine	30
Hasandede beyazi	6.38 ± 0.261	Wine	MED_EAST	NA	Admixture	30
Henab turki	6.34 ± 0.258	Table	MED_EAST	EAST_Table	NA	30
Henab Turki	6.19 ± 0.43	Table	BALKANS	NA	NA	30
Heptakilo	6.48 ± 0.21	Wine	BALKANS	EAST_Table	Admixture	30
Heunisch rot	6.02 ± 0.274	Wine	EUR_WEST	Admixture	Admixture	30
Heunisch schwarz	5.48 ± 0.238	Wine	EUR_WEST	Admixture	WCEUR_Wine	30
Horoz karasi	7.21 ± 0.355	Mixed	MED_EAST	NA	Admixture	30
Humagne blanc	6.11 ± 0.318	Wine	EUR_WEST	Admixture	NA	30
Hunisa	8.34 ± 0.369	Table	ASIA_CENT	EAST_Table	NA	30
Hweidi	6.93 ± 0.491	Table	MED_EAST	EAST_Table	Admixture	30
Imeruli shavi	5.89 ± 0.213	Wine	MED_EAST	NA	NA	30
Irsai Oliver	6.19 ± 0.334	Mixed	BALKANS	Admixture	Admixture	30
Ispisari	7.05 ± 0.294	Table	BALKANS	NA	EAST_Table	30
Italia	6.84 ± 0.322	Table	EUR_ITAL	Admixture	NA	30
Ithaki	5.94 ± 0.378	Table	BALKANS	Admixture	Admixture	30
Jahafi	6.82 ± 0.286	Table	MED_EAST	NA	EAST_Table	30
July Muscat	7.22 ± 0.264	Table	NEW_WORLD	Admixture	Admixture	30
Kadarka = Gamza	5.9 ± 0.391	Wine	BALKANS	BALK_Wine	BALK_Wine	30
Kadin Barmak	7.96 ± 0.323	Table	MED_EAST	NA	Admixture	30
Kalecik siyahi	6.94 ± 0.403	Wine	MED_EAST	NA	Admixture	30
Kamuri Shavi	6.69 ± 0.336	Wine	MED_EAST	NA	NA	30
Kara ouzume Aschkhabadsky	6.69 ± 0.226	Mixed	ASIA_CENT	Admixture	EAST_Table	30
Karasakiz	6.9 ± 0.266	Wine	MED_EAST	NA	Admixture	30
Karolowska = Xinomavro	5.89 ± 0.265	Wine	NA	NA	NA	30
Kartsiotis	6.9 ± 0.291	Wine	BALKANS	BALK_Wine	BALK_Wine	30
Karul	7.11 ± 0.423	Table	NA	NA	NA	30
Kasoufi de la Bekaa	6.61 ± 0.303	Wine	MED_EAST	EAST_Table	Admixture	30
Katsacoulias	6.7 ± 0.273	Wine	BALKANS	NA	Admixture	30
Keçimemesi noir	6.99 ± 0.632	Table	NA	NA	NA	27
Kharistvala tetri	7.16 ± 0.369	Wine	MED_EAST	NA	NA	30
Khikhvi	6.63 ± 0.274	Wine	MED_EAST	Admixture	NA	28
Khindogny	6.12 ± 0.273	Wine	ASIA_CENT	Admixture	Admixture	30
Khoussaïné blanc	7.72 ± 0.676	Table	ASIA_CENT	EAST_Table	EAST_Table	27
Kibris	7.27 ± 0.443	NA	NA	NA	NA	30
Kincsem	6.07 ± 0.279	Mixed	BALKANS	NA	Admixture	30
Kirtsistela	5.99 ± 0.265	Wine	MED_EAST	NA	NA	30
Koïniariko	6.77 ± 0.335	Table	BALKANS	NA	Admixture	30
Kokkinostaphylo	6.26 ± 0.461	Wine	BALKANS	NA	NA	30
Koloshi	6.75 ± 0.183	Wine	MED_EAST	NA	NA	30
Königstraube	5.88 ± 0.21	Mixed	EUR_WEST	Admixture	Admixture	30
Kontegalo	8.58 ± 0.465	Table	BALKANS	EAST_Table	Admixture	30
Korithi mavro	6.62 ± 0.358	Mixed	BALKANS	NA	BALK_Wine	30
Koritsanos	5.67 ± 0.364	Wine	BALKANS	NA	NA	30
Kotsifali	6.89 ± 0.25	Wine	BALKANS	Admixture	Admixture	30
Kouroupitsa	6.23 ± 0.19	Wine	BALKANS	Admixture	Admixture	30
Kövidinka	5.56 ± 0.195	Wine	BALKANS	BALK_Wine	NA	30
Koz ouzioum	7.42 ± 0.28	Mixed	EUR_EAST	Admixture	EAST_Table	30
Krakhouna	6.55 ± 0.339	Mixed	MED_EAST	Admixture	NA	30
Kravi tzitzi	7.14 ± 0.276	Table	BALKANS	NA	ITACE_Table	30
Ladikino	6.1 ± 0.257	Wine	BALKANS	NA	Admixture	30
Lagrein	5.95 ± 0.287	Wine	EUR_ITAL	Admixture	Admixture	30
Lambrusco di Sorbara	7.44 ± 0.218	Wine	EUR_ITAL	WEST_Wine	Admixture	30
Lambrusco Marani	7.3 ± 0.359	Wine	EUR_ITAL	Admixture	Admixture	30
Lekuri kurdzeni	6.15 ± 0.205	Wine	MED_EAST	NA	NA	29
Len de l'El	6.13 ± 0.424	Wine	EUR_WEST	Admixture	NA	30
Lialmigui	7 ± 0.247	Mixed	EUR_EAST	Admixture	Admixture	30
Lignan	6.48 ± 0.45	Table	EUR_ITAL	Admixture	NA	30
Listan	5.59 ± 0.233	Mixed	EUR_IBER	NA	NA	30
Lisztes feher	6.01 ± 0.464	Wine	BALKANS	BALK_Wine	NA	30
Local black	5.45 ± 0.42	Wine	BALKANS	NA	NA	30
Loureiro	5.54 ± 0.186	Wine	EUR_IBER	Admixture	Admixture	30
Madeleine angevine Oberlin	6.93 ± 0.277	Table	EUR_WEST	Admixture	Admixture	30
Madeleine de Clermont	7.16 ± 0.21	Table	EUR_WEST	Admixture	NA	30
Madeleine salomon	5.94 ± 0.402	Table	EUR_ITAL	Admixture	NA	29
Magdeleine noire des Charentes	7.31 ± 0.351	Wine	EUR_WEST	WEST_Wine	WCEUR_Wine	30
Maingonnat 3 L 1	6.23 ± 0.262	Wine	MED_SOUTH	NA	IBER_WT	30
Malvar	5.75 ± 0.176	Mixed	EUR_IBER	IBER_Wine	IBER_WT	30
Malvasia del Chianti	5.58 ± 0.211	Wine	EUR_ITAL	NA	Admixture	30
Malvasia di Candia	6.39 ± 0.317	Wine	EUR_ITAL	NA	Admixture	30
Malvasia di Candia aromatica	6.81 ± 0.313	Wine	EUR_ITAL	NA	Admixture	30
Malvasia di Sardegna	6.2 ± 0.513	Wine	EUR_ITAL	Admixture	NA	30
Malvasia istriana	5.92 ± 0.308	Wine	BALKANS	Admixture	Admixture	30
Mancin	5.72 ± 0.319	Wine	EUR_WEST	WEST_Wine	WCEUR_Wine	30
Manda Gozu	7.59 ± 0.313	NA	NA	NA	NA	30
Manizi	7.33 ± 0.347	Table	BALKANS	NA	BALK_Wine	30
Manseng noir	6.18 ± 0.301	Wine	EUR_WEST	WEST_Wine	WCEUR_Wine	30
Maratheftiko	6.84 ± 0.343	Mixed	BALKANS	Admixture	Admixture	30
Matrassa	6.4 ± 0.585	Wine	MED_EAST	Admixture	Admixture	30
Maturana blanca	6.72 ± 0.29	Wine	EUR_IBER	Admixture	Admixture	30
Mauzac	6.36 ± 0.339	Wine	EUR_WEST	Admixture	WCEUR_Wine	30
Mavrodaphni	6.52 ± 0.301	Wine	BALKANS	NA	Admixture	30
Mavrostypho	6.54 ± 0.253	Wine	BALKANS	NA	Admixture	30
Mavrud	6.88 ± 0.277	Wine	BALKANS	NA	BALK_Wine	30
Mavrud Varnenski	5.68 ± 0.162	Wine	BALKANS	NA	NA	30
Medouar	6.97 ± 0.331	NA	MED_EAST	EAST_Table	EAST_Table	30
Melon	6.09 ± 0.261	Wine	EUR_WEST	NA	NA	30
Mencia	5.63 ± 0.195	Wine	EUR_IBER	Admixture	Admixture	30
Merlot	6.27 ± 0.222	Wine	EUR_WEST	Admixture	NA	30
Merlot blanc	6.35 ± 0.258	Wine	EUR_WEST	NA	WCEUR_Wine	30
Merlot gris	6.18 ± 0.354	Wine	EUR_WEST	NA	NA	30
Merseguera	6.02 ± 0.265	Wine	EUR_IBER	IBER_Wine	IBER_WT	30
Meslier Saint−François	5.54 ± 0.663	Wine	EUR_WEST	Admixture	NA	30
Meunier	5.91 ± 0.282	Wine	EUR_WEST	NA	NA	30
Miguel de Arco	6.15 ± 0.232	Wine	EUR_IBER	IBER_Wine	Admixture	30
Misket Rusenski	7.39 ± 0.464	Table	BALKANS	Admixture	NA	30
Mission	5.79 ± 0.204	Wine	NEW_WORLD	IBER_Wine	Admixture	30
Mondeuse	6.97 ± 0.292	Wine	EUR_WEST	WEST_Wine	WCEUR_Wine	30
Mondeuse blanche	6.77 ± 0.345	Wine	EUR_WEST	Admixture	WCEUR_Wine	30
Monemvassia	5.57 ± 0.319	Mixed	BALKANS	NA	NA	30
Moravia agria	5.97 ± 0.273	Wine	EUR_IBER	Admixture	NA	30
Morrastel	6.74 ± 0.285	Wine	EUR_WEST	Admixture	NA	30
Moscato di Terracina	6.84 ± 0.311	Mixed	EUR_ITAL	Admixture	NA	30
Mourvèdre	6.18 ± 0.419	Wine	EUR_IBER	Admixture	Admixture	30
Mskhali	6.92 ± 0.262	Mixed	MED_EAST	Admixture	Admixture	30
Muscat à petits grains blancs	5.97 ± 0.312	Wine	BALKANS	Admixture	NA	30
Muscat à petits grains roses	5.85 ± 0.267	Wine	BALKANS	NA	NA	30
Muscat d'Istamboul	6.23 ± 0.229	Wine	MED_SOUTH	Admixture	Admixture	30
Muscat d'Ouzbekistan	7.29 ± 0.286	Table	EUR_EAST	NA	NA	30
Muscat de Hambourg	7.45 ± 0.307	Table	NA	NA	NA	30
Muscat Ottonel	6.23 ± 0.244	Wine	EUR_WEST	Admixture	Admixture	30
Muscat vörösmarty	6.91 ± 0.379	Table	BALKANS	NA	NA	30
Müsküle	7.08 ± 0.373	Table	MED_EAST	Admixture	Admixture	30
Mustos feher	6.71 ± 0.228	Wine	BALKANS	NA	BALK_Wine	30
Nassau	5.59 ± 0.292	Wine	EUR_EAST	Admixture	Admixture	30
Nebbiolo	6.95 ± 0.208	Wine	EUR_ITAL	NA	NA	30
Negra molle = Mollar cano	6.44 ± 0.283	Wine	EUR_IBER	NA	NA	30
Négret castrais	6.09 ± 0.294	Wine	EUR_WEST	Admixture	Admixture	30
Négret pounjut	6.21 ± 0.324	Wine	EUR_WEST	Admixture	WCEUR_Wine	30
Négrette	5.97 ± 0.37	Wine	EUR_WEST	NA	NA	30
Negru virtos	6.4 ± 0.301	Wine	BALKANS	BALK_Wine	BALK_Wine	30
Nehelescol	7.01 ± 0.338	Table	MED_EAST	Admixture	NA	30
Nerello cappuccio	7.06 ± 0.228	Wine	EUR_ITAL	Admixture	Admixture	30
Nerello mascalese	6.41 ± 0.472	Wine	EUR_ITAL	NA	Admixture	30
Nero grosso	6.64 ± 0.243	Wine	EUR_ITAL	Admixture	Admixture	30
Nuragus	6.53 ± 0.314	Wine	EUR_ITAL	Admixture	NA	30
Obeidi	6.7 ± 0.331	Table	MED_EAST	Admixture	Admixture	30
Odem	6.84 ± 0.34	Table	MED_EAST	EAST_Table	EAST_Table	30
Odjaleschi	6.41 ± 0.217	Wine	MED_EAST	Admixture	Admixture	30
Ohanes	7.66 ± 0.364	Table	EUR_IBER	Admixture	IBER_WT	30
Okhtoura	6.16 ± 0.262	Wine	MED_EAST	NA	NA	30
Öküzgözü	6.94 ± 0.349	Table	MED_EAST	EAST_Table	NA	30
Olivette blanche	7.24 ± 0.181	Table	NA	Admixture	NA	30
Orbeluri ojaleshi	6.28 ± 0.353	Wine	MED_EAST	NA	NA	30
Orbois	6.97 ± 0.46	Wine	EUR_WEST	NA	WCEUR_Wine	30
Otskhanuri saphere	6.26 ± 0.453	Wine	MED_EAST	NA	NA	30
Pagadebiti	6.53 ± 0.384	Wine	EUR_WEST	Admixture	Admixture	30
Pamid Tcher	6.14 ± 0.252	Wine	BALKANS	NA	NA	30
Papazkarasi	6.1 ± 0.372	Wine	MED_EAST	BALK_Wine	NA	30
Peck	7.12 ± 0.403	Table	MED_SOUTH	NA	Admixture	30
Pedro Ximénez	6.14 ± 0.267	Wine	EUR_IBER	IBER_Wine	NA	30
Pehlivan kara = Papigi kara	6.52 ± 0.307	Table	MED_EAST	NA	BALK_Wine	30
Peikani	7.63 ± 0.344	Table	ASIA_CENT	Admixture	EAST_Table	30
Pembe Gemre	6.92 ± 0.313	Table	MED_EAST	NA	NA	30
Persan	6.76 ± 0.313	Wine	EUR_WEST	WEST_Wine	WCEUR_Wine	30
Petit Manseng	6.03 ± 0.314	Wine	EUR_WEST	WEST_Wine	WCEUR_Wine	30
Petit Meslier	7.38 ± 0.495	Wine	EUR_WEST	NA	NA	30
Petit verdot	5.97 ± 0.267	Wine	EUR_WEST	WEST_Wine	WCEUR_Wine	30
Petrokoritho mavro	5.82 ± 0.307	Wine	BALKANS	NA	BALK_Wine	30
Peurion	5.61 ± 0.311	Wine	EUR_WEST	NA	WCEUR_Wine	30
Peygamber üzümü	7.6 ± 0.371	Table	MED_EAST	EAST_Table	Admixture	30
Picadillo	5.32 ± 0.278	Wine	EUR_IBER	Admixture	IBER_WT	30
Pinot blanc	6.26 ± 0.221	Wine	EUR_WEST	NA	NA	30
Pinot gris	6.25 ± 0.406	Wine	EUR_WEST	NA	NA	30
Pinot noir	6.87 ± 0.421	Wine	EUR_WEST	WEST_Wine	WCEUR_Wine	30
Pinotage	5.25 ± 0.246	Wine	NEW_WORLD	WEST_Wine	Admixture	30
Piquepoul blanc	6 ± 0.276	Wine	EUR_WEST	NA	WCEUR_Wine	30
Planta nova	5.96 ± 0.245	Mixed	EUR_IBER	Admixture	Admixture	30
Platyracho	6.03 ± 0.283	Wine	BALKANS	Admixture	BALK_Wine	30
Plavina crna	6.13 ± 0.34	Wine	BALKANS	Admixture	BALK_Wine	30
Portugais bleu	6.75 ± 0.281	Mixed	EUR_WEST	Admixture	Admixture	30
Posip bijeli	6.89 ± 0.423	Wine	BALKANS	Admixture	Admixture	30
Pougayen	7.18 ± 0.245	Wine	EUR_WEST	Admixture	WCEUR_Wine	30
Précoce Bousquet = Honigler	5.59 ± 0.257	Wine	EUR_WEST	NA	NA	30
Prieto picudo	6.4 ± 0.224	Wine	EUR_IBER	Admixture	NA	30
Primitivo	5.95 ± 0.246	Wine	EUR_ITAL	Admixture	Admixture	30
Prokupac	6.22 ± 0.278	Wine	BALKANS	BALK_Wine	BALK_Wine	30
Prunelard	6.93 ± 0.316	Wine	EUR_WEST	NA	WCEUR_Wine	30
Qisi	6.46 ± 0.354	Table	MED_EAST	NA	NA	30
Quebranta	6.28 ± 0.274	Wine	NEW_WORLD	NA	Admixture	30
Quiebratinajas	6.02 ± 0.376	Mixed	EUR_IBER	Admixture	IBER_WT	30
Rabigato	6.19 ± 0.222	Wine	EUR_IBER	Admixture	Admixture	30
Ramisco	6.68 ± 0.246	Wine	EUR_IBER	Admixture	NA	30
Rannii Vira	8.07 ± 0.266	Table	EUR_EAST	EAST_Table	EAST_Table	30
Rassegui	6.79 ± 0.338	Mixed	MED_SOUTH	Admixture	NA	30
Razachie rosie	7.97 ± 0.399	Table	BALKANS	Admixture	BALK_Wine	30
Razaki pembe	6.87 ± 0.271	Table	MED_EAST	NA	Admixture	30
Red globe	7.95 ± 0.486	Table	NEW_WORLD	Admixture	Admixture	30
Ribolla gialla	5.44 ± 0.279	Mixed	EUR_ITAL	NA	Admixture	30
Riesling	6.01 ± 0.301	Wine	EUR_WEST	Admixture	WCEUR_Wine	30
Riesling italico	6.33 ± 0.291	Wine	EUR_ITAL	Admixture	NA	30
Rivairenc = Aspiran noir	6.28 ± 0.295	Mixed	EUR_WEST	Admixture	Admixture	30
Rkatsiteli	6.71 ± 0.362	Mixed	MED_EAST	NA	NA	30
Roditis	5.59 ± 0.311	Mixed	BALKANS	NA	Admixture	30
Rosa menna di vacca	7.28 ± 0.217	Table	BALKANS	Admixture	Admixture	30
Roter veltliner	5.51 ± 0.249	Wine	EUR_WEST	NA	NA	30
Roublot	6.04 ± 0.447	Wine	EUR_WEST	Admixture	WCEUR_Wine	30
Roumi noir	6.43 ± 0.326	Table	MED_EAST	Admixture	Admixture	30
Roussaïtis	6.39 ± 0.326	Wine	BALKANS	NA	BALK_Wine	30
Roussanne	5.46 ± 0.292	Wine	EUR_WEST	NA	Admixture	30
Rtskhili	6.36 ± 0.399	Wine	MED_EAST	NA	NA	30
Ruby cabernet	6.1 ± 0.303	Wine	NEW_WORLD	Admixture	Admixture	30
Rufete	6.25 ± 0.372	Wine	EUR_IBER	Admixture	Admixture	30
Ruzevina (Jelaska)	5.98 ± 0.341	Mixed	BALKANS	Admixture	BALK_Wine	30
Sahebi	7.92 ± 0.344	Table	ASIA_CENT	NA	NA	29
Sakmevela	6.51 ± 0.382	Wine	MED_EAST	NA	NA	30
Salba	5.94 ± 0.378	Table	NEW_WORLD	NA	Admixture	30
Samoriau	6.72 ± 0.277	Wine	EUR_WEST	NA	WCEUR_Wine	30
Santanera	5.57 ± 0.275	Wine	EUR_IBER	Admixture	IBER_WT	30
Sapena	6.1 ± 0.318	Wine	MED_EAST	NA	NA	30
Saperavi	5.7 ± 0.211	Wine	MED_EAST	Admixture	NA	30
Sauvignon	7.61 ± 0.318	Wine	EUR_WEST	NA	NA	30
Savagnin blanc = Weisser Traminer	6.33 ± 0.295	Wine	EUR_WEST	WEST_Wine	WCEUR_Wine	30
Savagnin rose	6.07 ± 0.251	Wine	EUR_WEST	NA	NA	30
Savvatiano	5.5 ± 0.288	Wine	BALKANS	Admixture	Admixture	30
Shavkapito	6.41 ± 0.229	Wine	MED_EAST	NA	NA	30
Siah	5.71 ± 0.477	Mixed	ASIA_CENT	Admixture	EAST_Table	26
Sideritis	6.06 ± 0.204	Table	BALKANS	NA	NA	30
Siegerrebe	7.14 ± 0.234	Wine	EUR_WEST	Admixture	Admixture	30
Sliva	6.84 ± 0.422	Table	BALKANS	NA	Admixture	30
Soïaki	6.7 ± 0.429	Mixed	ASIA_CENT	Admixture	EAST_Table	30
Sorok Let Oktiabria	5.98 ± 0.259	Wine	EUR_EAST	Admixture	Admixture	30
Sousão	5.88 ± 0.226	Wine	EUR_IBER	WEST_Wine	Admixture	30
Staphidostaphylo	6.09 ± 0.299	Wine	BALKANS	NA	BALK_Wine	30
Stavroto	6.65 ± 0.238	Wine	BALKANS	Admixture	Admixture	30
Steinschiller weisser	6.32 ± 0.199	Wine	BALKANS	Admixture	Admixture	30
Sultanine monococco	7.36 ± 0.289	Table	ASIA_CENT	NA	NA	30
Sylvaner	7.92 ± 0.422	Wine	EUR_WEST	Admixture	Admixture	30
Syrah	5.47 ± 0.257	Wine	EUR_WEST	WEST_Wine	WCEUR_Wine	30
Tachtas	5.93 ± 0.212	Mixed	BALKANS	EAST_Table	Admixture	30
Tagobi	7.84 ± 0.455	Mixed	ASIA_CENT	Admixture	EAST_Table	30
Tavkveri	6.49 ± 0.39	Wine	MED_EAST	Admixture	EAST_Table	30
Tavtsitela	6.69 ± 0.272	NA	MED_EAST	NA	NA	30
Teinturier	5.94 ± 0.396	Wine	EUR_WEST	WEST_Wine	NA	30
Tempranillo	6.45 ± 0.178	Wine	EUR_IBER	NA	IBER_WT	30
Terret noir	6.06 ± 0.362	Wine	EUR_WEST	NA	NA	30
Tibouren	6.71 ± 0.483	Mixed	EUR_WEST	Admixture	NA	30
Tinta Barroca	5.68 ± 0.21	Wine	EUR_IBER	Admixture	Admixture	30
Tinto cao	6.53 ± 0.236	Wine	EUR_IBER	Admixture	Admixture	30
Torrontès riojano	6.11 ± 0.333	Wine	NEW_WORLD	Admixture	ITACE_Table	30
Trebbiano spoletino	6.31 ± 0.407	Wine	EUR_ITAL	Admixture	Admixture	30
Tressot	5.64 ± 0.291	Wine	EUR_WEST	WEST_Wine	NA	30
Troyen	6.31 ± 0.307	Wine	EUR_WEST	NA	WCEUR_Wine	30
Tsapourno	6.32 ± 0.246	NA	NA	NA	NA	30
Tsitsa kaprei	6.92 ± 0.321	Table	BALKANS	Admixture	NA	30
Tsitska	6.31 ± 0.215	Wine	MED_EAST	Admixture	Admixture	30
Tskobila	6.12 ± 0.27	Wine	MED_EAST	NA	NA	30
Tsolikouri	6.55 ± 0.253	Wine	MED_EAST	Admixture	NA	30
Tsulikidzis tetra	6.35 ± 0.282	Wine	MED_EAST	NA	NA	30
Turfanda	6.36 ± 0.398	Table	NA	NA	NA	30
Turki	6.86 ± 0.246	Table	MED_SOUTH	Admixture	Admixture	30
Ubakluri	6.11 ± 0.245	Wine	MED_EAST	NA	NA	30
Ugni blanc	7.89 ± 0.299	Mixed	EUR_WEST	NA	NA	30
Urmi dinka	6.05 ± 0.32	Wine	BALKANS	BALK_Wine	BALK_Wine	30
Uva di Trojanero	5.79 ± 0.225	Wine	EUR_ITAL	Admixture	Admixture	30
Vaftra	6.54 ± 0.277	Wine	BALKANS	NA	Admixture	30
Valenci blanco	6.28 ± 0.213	Table	EUR_IBER	IBER_Wine	NA	30
Variété d'oasis Bou Chemma 46	5.5 ± 0.338	Table	MED_SOUTH	Admixture	NA	27
Varnenska Gimza	6.56 ± 0.342	Wine	BALKANS	BALK_Wine	BALK_Wine	30
Verdea noir	6.19 ± 0.258	Mixed	EUR_ITAL	Admixture	Admixture	30
Verdeca	7.15 ± 0.284	Wine	BALKANS	Admixture	NA	30
Verdelho tinto	7.87 ± 0.431	Wine	EUR_IBER	Admixture	Admixture	30
Verdzami	5.47 ± 0.162	NA	NA	NA	NA	30
Verjus	7.38 ± 0.329	Table	EUR_WEST	Admixture	NA	30
Vermentino	6.16 ± 0.425	Mixed	EUR_WEST	NA	NA	30
Vernaccia di San Gimignano	6.36 ± 0.406	Wine	EUR_ITAL	Admixture	Admixture	30
Vertkvichalis tetri	6.24 ± 0.3	Wine	MED_EAST	NA	NA	30
Vertzami = Lefkada	6.61 ± 0.273	Wine	BALKANS	NA	NA	30
Vespolina	7.22 ± 0.266	Wine	EUR_ITAL	NA	Admixture	30
Victoria	7.49 ± 0.414	Table	BALKANS	Admixture	Admixture	30
Vidiano	6.08 ± 0.285	Wine	BALKANS	Admixture	Admixture	30
Vilana	6.61 ± 0.319	Wine	BALKANS	NA	Admixture	30
Vintaghju	6.94 ± 0.385	Wine	EUR_WEST	Admixture	NA	30
Viognier	6.09 ± 0.224	Wine	EUR_WEST	NA	WCEUR_Wine	30
Voïdomatis	6.75 ± 0.258	Wine	BALKANS	Admixture	Admixture	30
Voskeat	5.98 ± 0.264	Wine	MED_EAST	Admixture	Admixture	30
Wildbacher blau	5.9 ± 0.261	Wine	EUR_WEST	Admixture	NA	30
Wildrebe von Bauer	6.41 ± 0.216	Wine	EUR_WEST	Admixture	Admixture	30
Xinisteri	5.83 ± 0.241	Mixed	BALKANS	Admixture	Admixture	30
Xinomavro	6.45 ± 0.235	Wine	BALKANS	NA	NA	30
Yag Uzumu	6.66 ± 0.42	Table	NA	NA	NA	30
Yaghasti	6.88 ± 0.392	Table	ASIA_CENT	NA	NA	30
Yapincak	5.84 ± 0.203	Mixed	MED_EAST	NA	Admixture	30
Zakatalis tetri	5.98 ± 0.282	Wine	MED_EAST	NA	NA	30
Zante blanc (Collection Ravaz)	5.44 ± 0.213	Wine	BALKANS	Admixture	NA	30
Zemoasa	6.73 ± 0.255	Wine	BALKANS	BALK_Wine	NA	30
Zweigelt	6.85 ± 0.415	Wine	EUR_WEST	Admixture	NA	30
